# Thiazolide Prodrug Esters and Derived Peptides: Synthesis and Activity

**DOI:** 10.1021/acsbiomedchemau.2c00083

**Published:** 2023-04-11

**Authors:** Andrew V. Stachulski, Jean-Francois Rossignol, Sophie Pate, Joshua Taujanskas, Jonathan A. Iggo, Rudi Aerts, Etienne Pascal, Sara Piacentini, Simone La Frazia, M. Gabriella Santoro, Lieven van Vooren, Liesje Sintubin, Mark Cooper, Karl Swift, Paul M. O’Neill

**Affiliations:** † Donnan and Robert Robinson Laboratories, Department of Chemistry, University of Liverpool, Liverpool L69 7ZD, U.K.; ‡ Romark Laboratories, L.C., Tampa, Florida 33609, United States; § Romark Belgium BVBA, Roosveld 6, 3400 Landen, Belgium; ∥ Department of Biology, University of Rome Tor Vergata, 00133 Rome, Italy; ⊥ Institute of Translational Pharmacology, CNR, Area della Ricerca di Roma 2, Via Fosso del Cavaliere, 00133 Roma, Italy; # Ardena Gent NV, Kleimor 4, 9030 Mariakerke, Belgium; ∇ Bio-Techne, Avonmouth, Bristol BS11 9QD, U.K.

**Keywords:** antiviral, prodrug, time-course NMR, rearrangement, influenza A, clinical trials

## Abstract

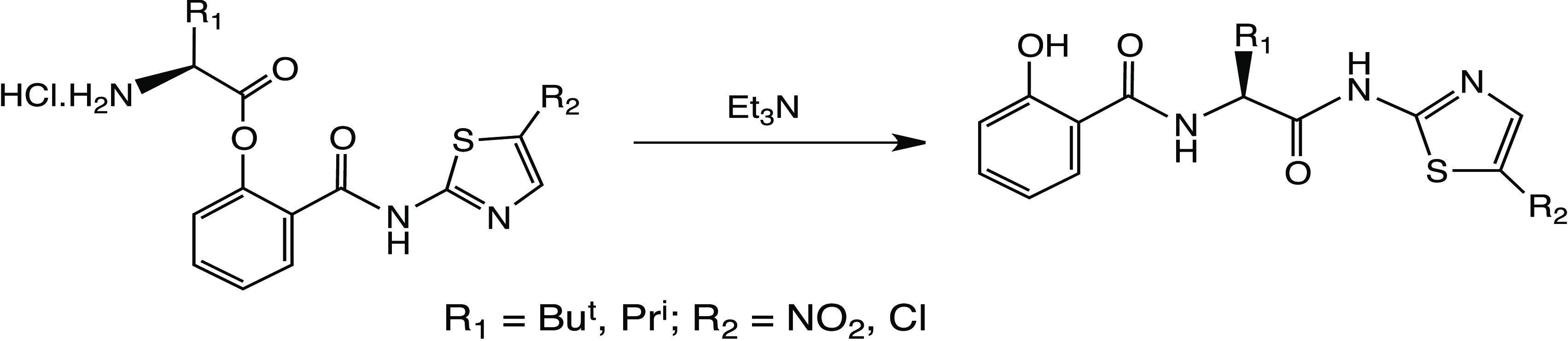

Amino acid ester prodrugs of the thiazolides, introduced to improve the pharmacokinetic parameters of the parent drugs, proved to be stable as their salts but were unstable at pH > 5. Although some of the instability was due to simple hydrolysis, we have found that the main end products of the degradation were peptides formed by rearrangement. These peptides were stable solids: they maintained significant antiviral activity, and in general, they showed improved pharmacokinetics (better solubility and reduced clearance) compared to the parent thiazolides. We describe the preparation and evaluation of these peptides.

## Introduction

The thiazolides,^1^ exemplified by nitazoxanide (NTZ) **1** and RM5038 **2**, are broad-spectrum antiviral agents with proven activity in cell line assays^2−5^ and clinical trials^6,7^ against a number of DNA and RNA viruses. Most recently, NTZ **1** has been shown to be effective in clinical trials against SARS-CoV-2, specifically in preventing progression of the disease from the mild to the severe phase.^8,9^ In the body, these *O*-acetates behave as simple prodrugs for the free phenols **3** and **4**, which are the effective circulating forms of the drugs in vivo.
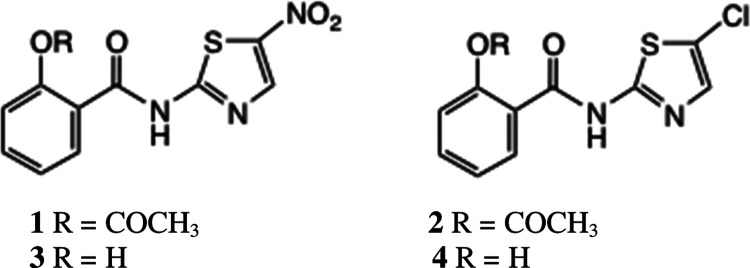


Nevertheless, the pharmacokinetic parameters of **1**, **2**, and other analogues, especially their low solubility, reduce their effectiveness toward respiratory viruses where a good circulating concentration of drug is important. Amino acid prodrugs have frequently been used to address this problem:^10−12^ consequently, we synthesized amino acid^13^ ester derivatives of **1** and **2**, specifically the *tert*-leucine derivatives **5a** and **6**.^14^ Here, the bulky *t*-butyl group was necessary to impart sufficient stability to the products; **5a** and **6** were stable for months at room temperature. In contrast, the corresponding valine esters (see below) decomposed at a significant rate at 20 °C.
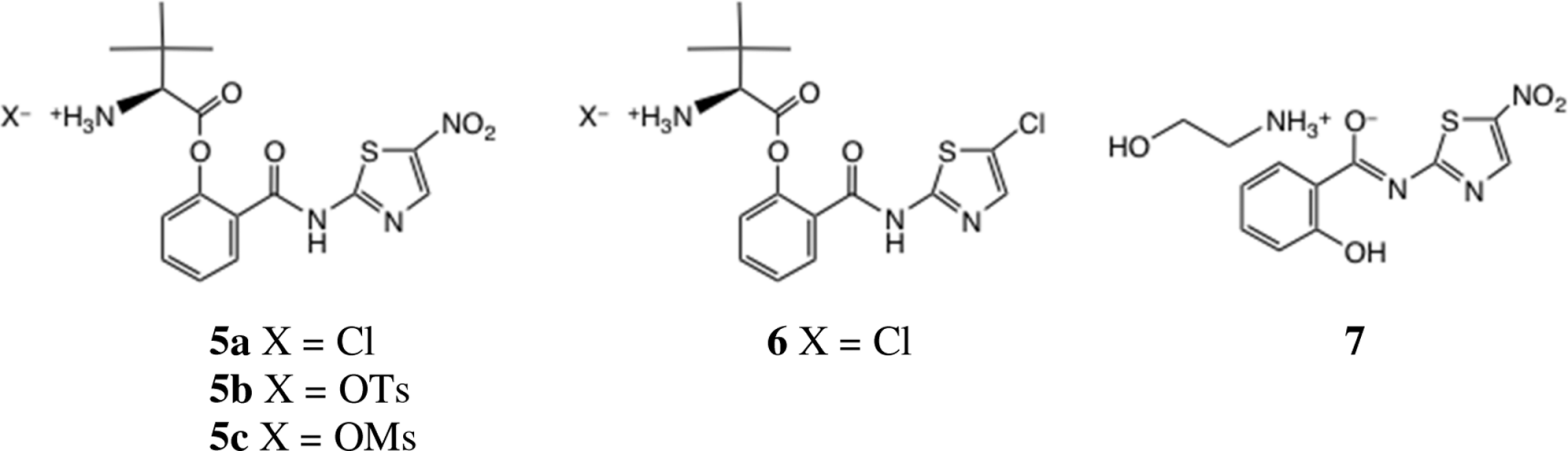


We obtained proof of concept: the absolute bioavailability of **5a**/**6** was 20–25%,^13^ as measured by oral bioavailability in rats, although the aqueous solubility of **5a** was still not high. By contrast, salt **6** had better solubility and could be recrystallized. An alternative approach, which we recently described,^15^ is offered by thiazolide amine salts such as **7**, which also possess superior pharmacokinetics to **1** and **2**, especially better solubility.

Since it proved difficult to obtain consistent formulations of **5a** as the HCl salt, we also prepared the corresponding tosylate^16^ and mesylate salts **5b** and **5c**. These were obtained microanalytically pure and, in contrast to **5a**, could be recrystallized without decomposition. It was still difficult to characterize such products by high-performance liquid chromatography (HPLC) analysis, however, as even at pH 5, solutions of these salts proved to be unstable, with apparently one major product being formed. We now report on the characterization and biological evaluation of the rearrangement products.

## Results and Discussion

### NMR Studies

**Figure 1:**
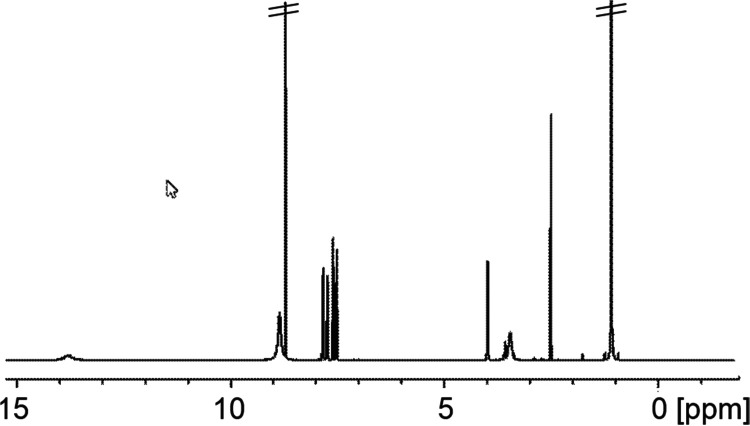
^1^H NMR spectrum of pure **5a** in *d*_6_-DMSO.

**Figure 2:**
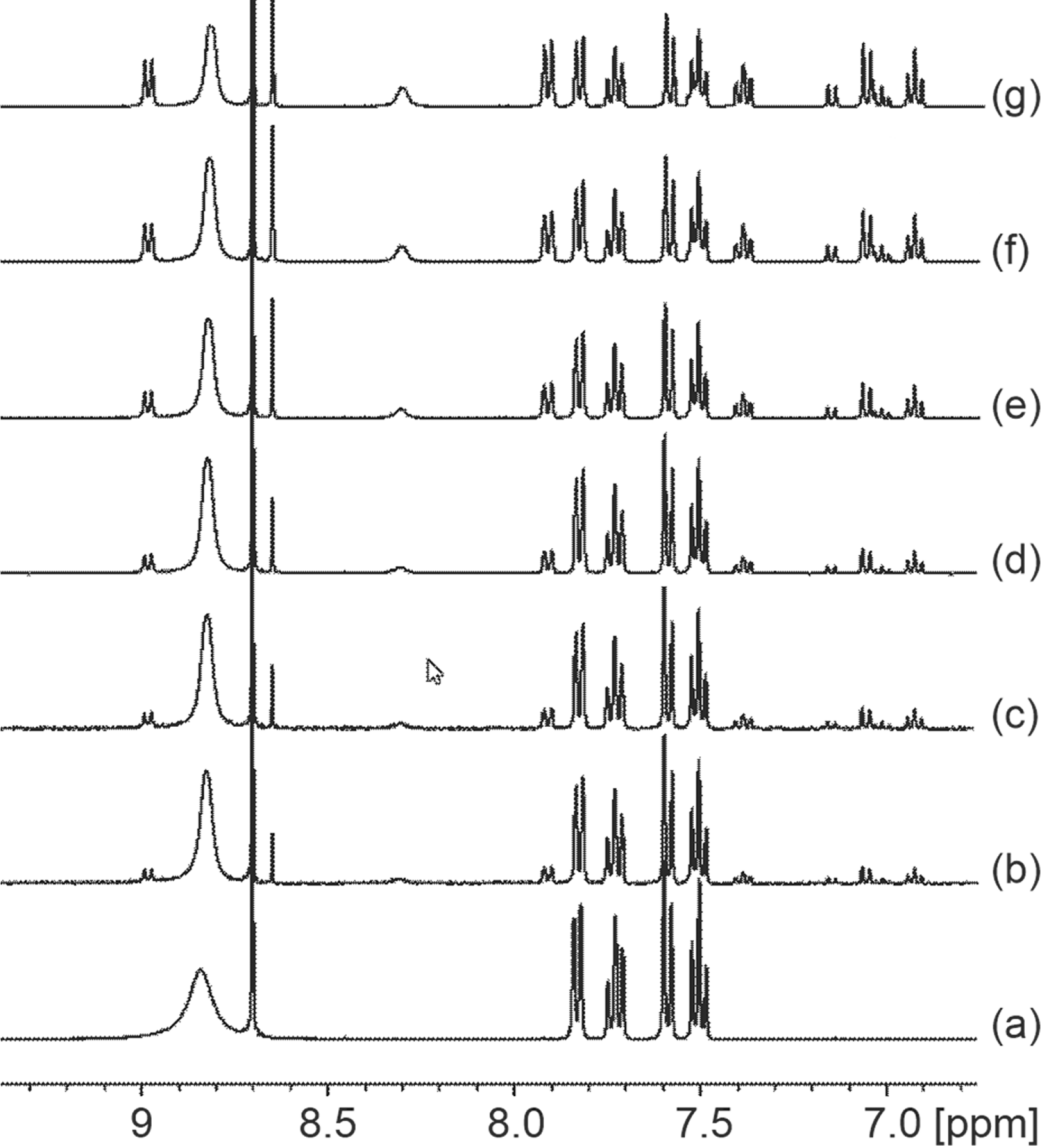
Time-course ^1^H NMR spectra of the aromatic region of **5a** in *d*_6_-DMSO + 10%D_2_O. Evolution with time over an 18 day period at 298–313 K. The sample was held in an isothermal bath at a specified temperature between measurements. Spectra were recorded at the temperature stated in each case. (a) *t* = zero, 298 K; (b) *t* = 1 week, 308 K; (c) *t* = 8 days, 308 K; (d) *t* = 9 days, 308 K; (e) *t* = 11 days, 313 K; (f) *t* = 14 days, 313 K; (g) *t* = 18 days, 313 K.

**Figure 3:**
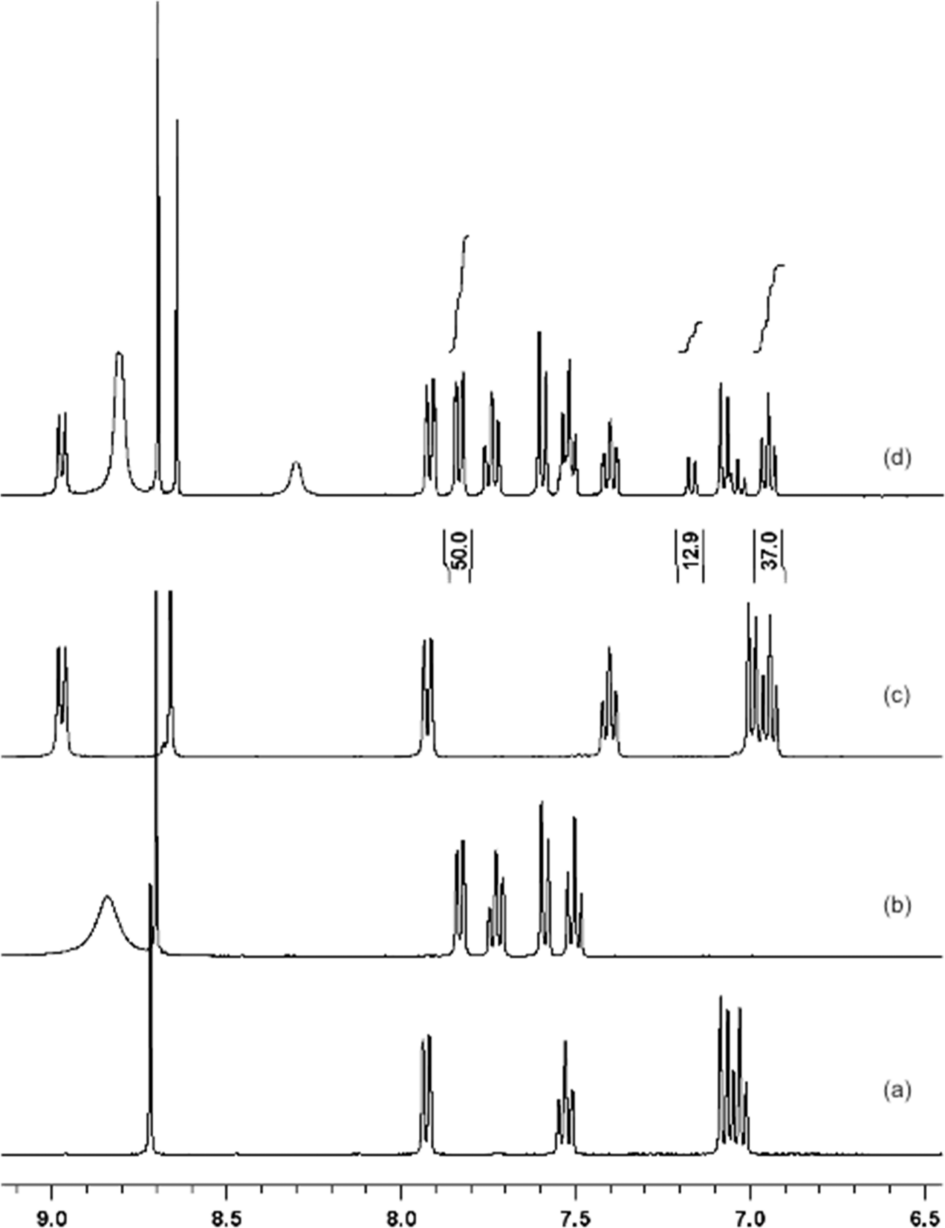
Assignment and integration of the final time-point spectrum. The figure shows the Ar region protons corresponding to: (a) tizoxanide **3**, (b) prodrug ester **5a**, (c) rearranged product **8**, and (d) the final time-point spectrum showing by integration a virtually 50% loss of **5a**.

We studied the aqueous stability further by NMR, Figures 1 and 2. Solutions of **5a** in *d*_6_-DMSO with 10% added D_2_O held at 25–40 °C showed a steady decomposition: a small part of this behavior could be attributed to direct hydrolysis, regenerating **3**, but a new major product was clearly being generated even without an added base. In the case of valacyclovir, which we originally used as a model for **5a** and **6**, it was known that some hydrolysis occurred in vivo.^17^ Clearly, at the 18 day time point [Figure 2g], the rearrangement and hydrolysis products accounted for ∼50% of the total analyte. In Figure 3, the aryl region is integrated and compared with the spectra of **5a**, **3**, and **8** (see below).

### Base-Catalyzed Rearrangement

Using initially preparative HPLC, a sample of the degradation product from **5a** was purified and characterized. In fact, it was easier to obtain this new product by briefly stirring a suspension of **5a** in tetrahydrofuran (THF) with one equivalent of triethylamine at 20 °C (0.5 h) followed by chromatography. This product, readily obtained in a microanalytically pure state, was shown to be the pseudotripeptide **8**, corresponding to an insertion of the amino acid unit into the amide bond of **5a**.
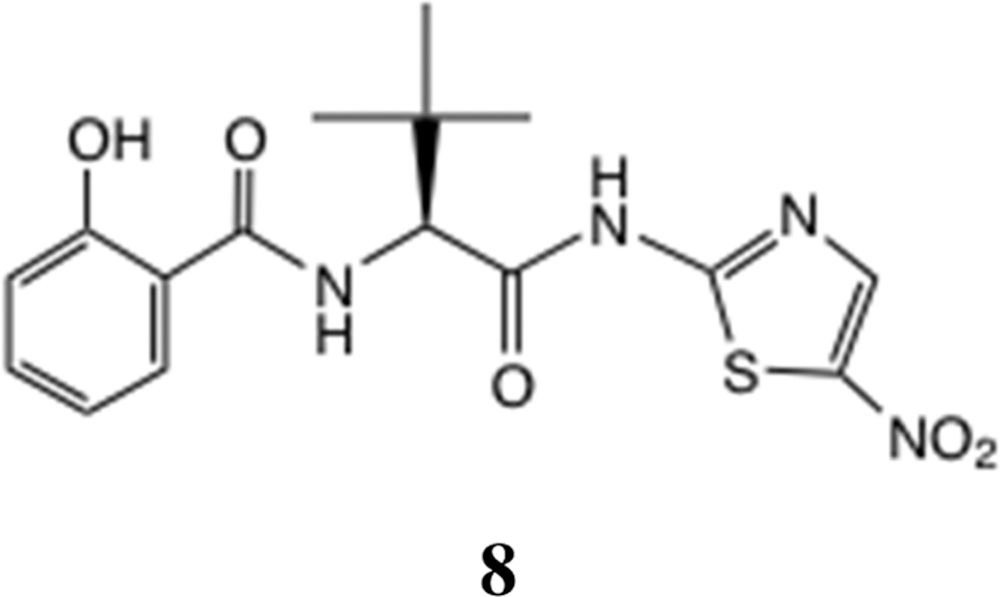


This kind of rearrangement has a precedent. Thus, Vinsova et al. obtained similar tripeptides derived from salicylanilides, e.g., **9** from amino acid ester **10**.^18,19^ A mechanism was proposed, and a byproduct **11** was isolated, characterized by X-ray crystallography, corresponding to dehydration of an imidazolinone intermediate along the reaction pathway. In the Supporting Information, we give a plausible mechanism for the rearrangement of **5a** to **8** and highlight the “cyclol” intermediate, which could lead to a byproduct akin to **11**.
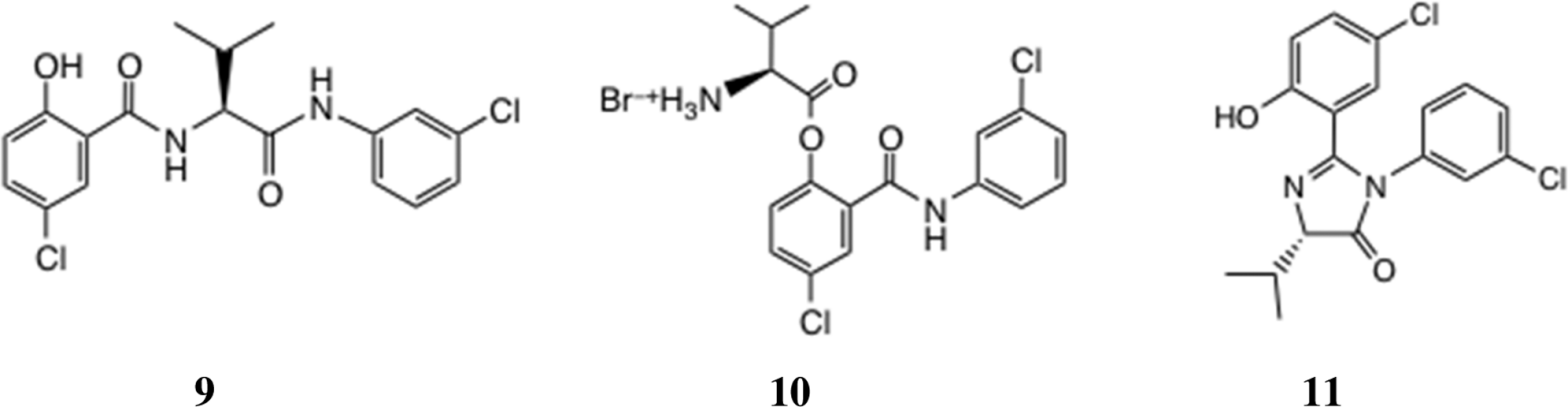


In this sequence, the precursors of esters **10**, which were N-protected with the benzyloxycarbonyl (Z) group, were shown to have antimicrobial activity especially against some fungal strains.^20^ A later paper,^21^ which did not cite the work of the Vinsova group, reported tripeptide-like compounds **12**, similar to **9** and formally related to niclosamide.^22,23^ Such derivatives were shown to have useful activity against adenovirus: the screened analogues were prepared by linear peptide synthesis from the appropriate amino acid anilides.
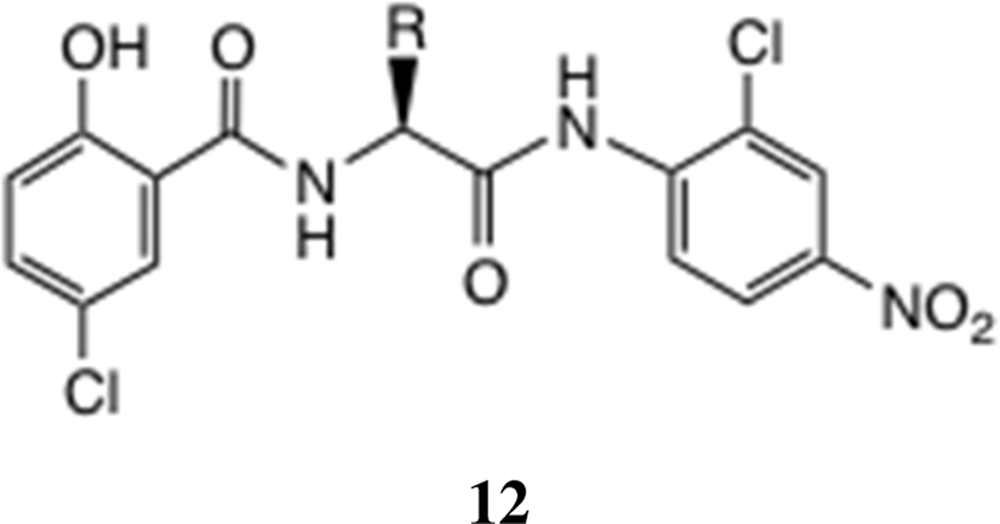


The history of the (O-acyl)salicyloyl amide rearrangement actually dates back at least to the 1950s. Thus, Brenner et al. reported^24,25^ the synthesis of salicyloyl di- and tripeptides such as **13** from salicylic acid derivatives by a sequence of O-acylation, deprotection, and basification, using Z-protected amino acids. An *O*- to *N*-acetyl transfer in a salicylamide derivative was reported by Titherley and Hicks in 1905.^26^ In our series, and Vinsova’s, we believe the *N*-aryl/heteroaryl group in **5a** and **10** may facilitate this rearrangement by increasing the acidity of the NH proton.
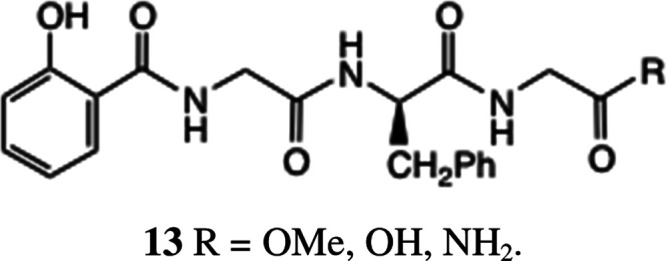


### Synthesis and Evaluation of Peptidic Analogues

**Scheme 1:**
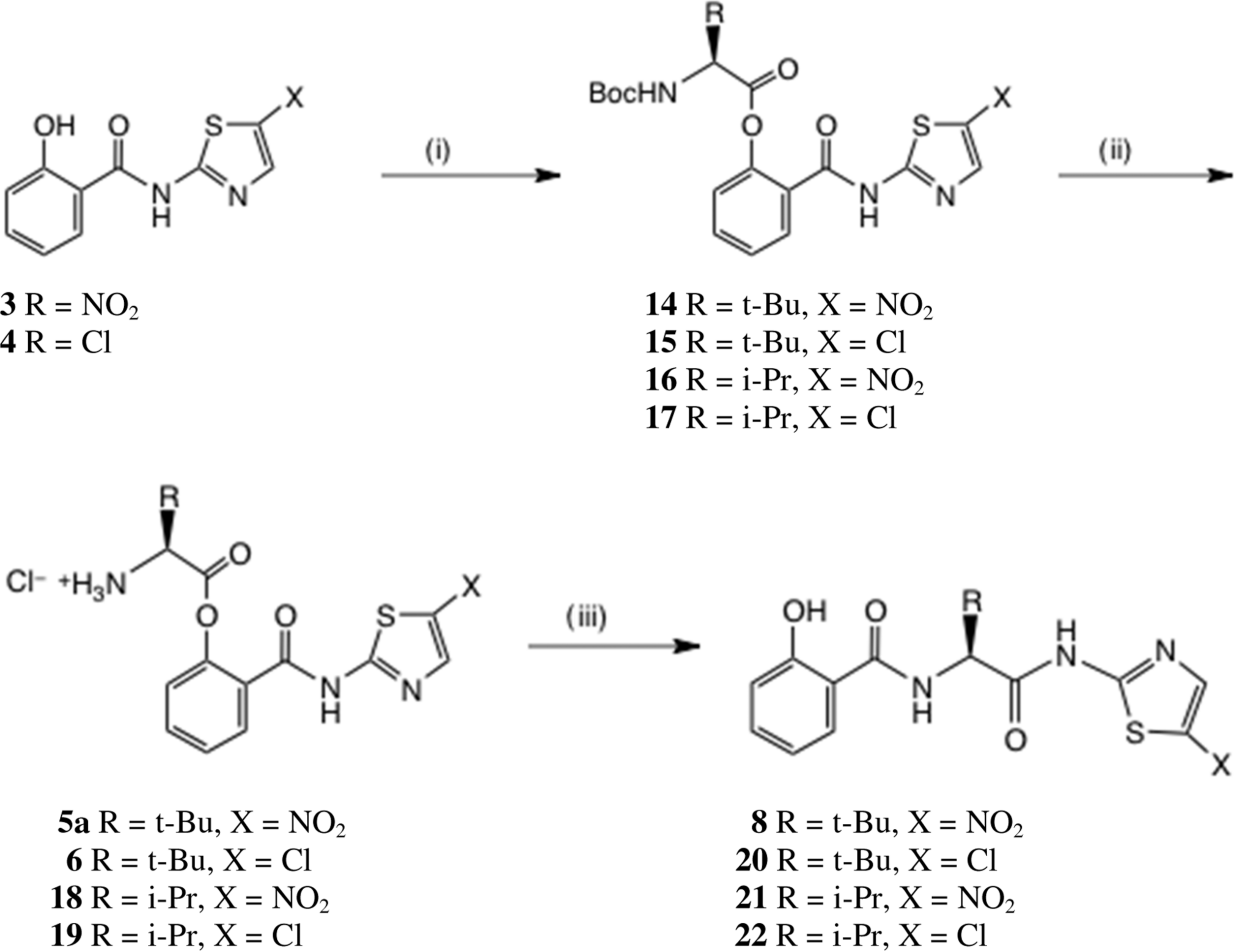
General Synthesis of Aminothiazolyl Tripeptides Conditions: (i) EDC, Boc-amino acid, DMAP, THF, 0–20 °C, 54–67%; (ii) 4 M HCl-dioxan, CH_2_Cl_2_, 20 °C, 78–94%; (iii) Et_3_N, THF, 0–20 °C, 52–76%.

We naturally investigated the activity and pharmacokinetic parameters of **8** and analogues, not least because any activity in tripeptides such as **8** could be partly responsible for the activity of **5a** and **6**. Since the work of Sanchez-Cespedes et al.^21^ showed that the valine analogue of their niclosamide-derived series was the most active against adenovirus in their studies, we also prepared the corresponding Val analogues of **3** and **4**. The general synthesis of all of the peptides is shown in Scheme 1.

Acylation of **3** or **4** with Boc-Val-OH or Boc-Tle-OH afforded esters **14**–**17** in 54–67% yield after chromatography. Deprotection with 4 M HCl-dioxan then gave the prodrug ester HCl salts **5a**, **6**, **18**, and **19** in good to excellent yields.^13^ Alternatively, in the case of **14**, the use of toluene *p*-sulfonic acid or methanesulfonic acid afforded the corresponding sulfonate salts **5b** and **5c**. The rearrangement of salts **5a**, **6**, **18**, and **19** to the tripeptides **8** and **20**–**22** was complete after treatment with Et_3_N in THF for 0.5 h at 20 °C: the weaker base *N*-methylmorpholine gave a considerably slower reaction. Rearrangement products **8** and **20**–**22** were produced together with some parent thiazolides **3** or **4**, which were removed by chromatography. In the case of the valine analogue **22**, we streamlined the synthesis. After acylation of **4** with Boc-Val-OH, unreacted **4** was removed by chromatography and the crude product **17** was immediately progressed by deprotection as above. The resulting HCl salt **19** was subjected to base-catalyzed rearrangement as above, and direct recrystallization of the product afforded tripeptide **22** in excellent purity.

The rearranged products were screened both for antiviral activity and for their DMPK properties, Table 1. In general terms, all four compounds **8** and **20**–**22** showed reduced clearance compared to the parent thiazolides, as well as better solubility for the 5-Cl derivatives. It is known that glucuronidation is the major clearance pathway for **3**(27) and **4**,^3^ but we have no detail regarding the metabolites of **8** and **20**–**22**. The significant decrease in clearance rates in hepatocytes, in contrast with the smaller effect on liver microsomes, is also consistent with a major glucuronidation metabolic pathway.

**Table 1:** Summarized DMPK and Antiviral Data for Thiazolides and Derived Peptides

compound	log *P*	solubility^a^	clearance RH μL/min/10^6^ cells	clearance HLM μL/min/mg	IC_50_^b^	IC_90_^b^	CC_50_^b^	SI^c^
**3**	1.8	33	244	50.2	2.3	26.4	>189	>82
**4**	2.9	48	>300	37.5	3.9	19.6	>196	>50
**8**	3.3	18	31.5	36.5	1.6	79	>132	>83
**20**	>3.5	388	75.4	42.4	>40.8^d^	>40.8	40.8	
**21**	2.9	>1000	67.7	55.9	13.7	>137	>137	>10
**22**	e	82	234	42.7	>42.4^d^	>42.4	42.4	

a Micromolar units at pH 7.4.

b Activities vs influenza A virus and cytotoxic concentrations (CC) are in μM. IC_50_ is 50% inhibitory concentration; IC90 is 90% inhibitory concentration; CC_50_ is 50% cytotoxic concentration.

c SI, selectivity index, viz. CC_50_/IC_50_.

d These compounds appear toxic.

e Failed to create analytical method.

Clearly, the nitro-tripeptide **8** retains good activity and a high cell safety index compared to the parent tizoxanide **3**. The corresponding valine analogue **21** is considerably less active and shows higher clearance figures; the influence of the *t*-butyl group is therefore also significant. We note that the earlier niclosamide-derived tripeptides^21^ featured only proteinogenic amino acids, with valine analogues as the closest parallel. By contrast, the chloro-tripeptides **20** and **22** were essentially inactive and appeared toxic under the assay conditions.

## Conclusions

Treatment of thiazolide amino acid esters **5a**, **6**, **18**, and **19**(13) with a base under anhydrous conditions leads to rapid rearrangement; the pseudotripeptides **8** and **20**–**22** can be isolated in good yields. An NMR study of **5a** in D_2_O/*d*_6_-DMSO showed that the same product was slowly formed under aqueous conditions, with some competing direct hydrolysis in both cases. The rearranged products of the nitro derivatives retained antiviral activity, e.g., **8** vs **5a** (whose activity is equivalent to **3**), and it is possible that **8** is partly responsible for the activity of **5a**. The nitro valine analogue **21** retained some antiviral activity. The DMPK data of **8** and **20**–**22** show reduced clearance in rat hepatocyte cells and, in some cases, in human liver microsomes compared to the prodrug esters.

While a similar rearrangement has been observed from niclosamide amino acid esters by other workers,^18,19^ the difference seen with the hindered amino acid *t*-Leu, leading to compounds **8** and **20**, is noticeable. Lower rat hepatocyte clearance is seen for these derivatives compared to the valine analogues **21** and **22**. Finally, chloro derivatives **20** and **22** are virtually inactive in the antiviral assay.

## Experimental Section

### General

Tizoxanide **3** and RM4848 **4** were supplied by Romark Pharmaceuticals. “Standard neutral workup” means that the product in organic solution was washed successively with 7% aq. citric acid, satd. aq. NaHCO_3_, and water. Organic extracts were finally washed with saturated brine and dried over anhydrous Na_2_SO_4_ prior to rotary evaporation at <30 °C. Moisture-sensitive reactions were carried out in anhydrous organic solvents (purchased from Sigma-Aldrich) under a N_2_ atmosphere. Reactions were monitored by analytical thin**-**layer chromatography using Merck Kieselgel 60 F_254_ silica plates and were viewed under ultraviolet (UV) or by staining with KMnO_4_ or iodine. Preparative flash column chromatography was performed on either VWR Prolabo silica gel or Sigma**-**Aldrich silica gel (particle size 40–63 Å). Mass spectra were obtained in either the electrospray mode (ES) with a Micromass LCT or the chemical ionization (CI) mode with a Micromass Trio 1000 using ammonia or methane as the carrier. Elemental analyses were performed by Mrs. Jean Ellis, of this department. ^1^H and ^13^C NMR spectra were obtained using a Bruker Avance-II instrument operating at 400.20 and 101.63 MHz for ^1^H and ^13^C, respectively; a Bruker Avance-IIIHD operating at 400.13 and 101.61 MHz for ^1^H and ^13^C, respectively; or a Bruker Avance-1 instrument operating at 400.03 and 101.59 MHz for ^1^H and ^13^C, respectively; using the stated solvent. Chemical shifts are reported in ppm (δ) relative to Me_4_Si. Coupling constants (*J*) are reported in Hz. Analytical HPLC traces were obtained using an Agilent column monitoring at 254 nm eluted with 5% MeCN-95% H_2_O containing 0.5% formic acid, at a flow rate of 1 mL/min. The purity of the tested compounds **8**, **20**, **21**, and **22** was >95% by HPLC.

#### (*S*)-2-[(5-Nitrothiazol-2-yl)carbamoyl]phenyl-2-((*tert*-butoxycarbonyl)amino)-3-methylbutanoate **16**

As previously described^13^ for the precursor of **5a**, a mixture of Boc-Val-OH (0.21 g, 0.97 mmol) and tizoxanide **3** (0.25 g, 0.94 mmol) was stirred at 20 °C in anhydrous THF (7.5 mL). *N*-Ethyl-*N*^′^-3-(dimethylamino)propyl carbodiimide·HCl (EDC; 0.19 g, 1 mmol) was added in one portion, followed immediately by 4-dimethylaminopyridine (DMAP; 0.12 g, 1 mmol). After 20 h, the mixture was filtered through Celite and the precipitate was washed with further THF and then diluted with ethyl acetate (25 mL). The combined filtrate and washings were washed with 7% aq. citric acid, saturated aq. NaHCO_3_, water, and brine and then dried over anhydrous Na_2_SO_4_ followed by evaporation, which afforded a yellow foam; this was chromatographed on silica gel, being applied in CHCl_3_ and eluted with EtOAc:hexane, 1:1. Appropriate fractions were combined and evaporated to afford the title compound **16** as an off-white solid (250 mg, 54%). Found: *m*/*z*, 487.1265. C_20_H_24_N_4_O_7_S.Na requires *m*/*z*, 487.1263; δ_H_ [400 MHz, CDCl_3_] 1.03, 1.12 (6H, 2d, *J* = 6.8 Hz, *Me*_2_CH), 1.40 (9H, s, Me_3_C), 2.35 (1H, m, Me_2_*CH*CH), 4.39 (1H, m, CH*CH*NH), 5.20 (1H, br m, NH), 7.40 (1H, d, *J* = 8.0 Hz, ArH), 7.45 (1H, t, *J* = 8.0 Hz, ArH), 7.67 (1H, t, *J* = 8.0 Hz, ArH), 8.06 (1H, d, *J* = 8.0 Hz, ArH), 8.18 (1H, s, thiazole 4-H), and 11.10 (1H, br s, NH); δ_C_ 17.9, 19.3, 28.2, 30.4, 59.7, 80.7, 123.4, 124.2, 126.7, 130.9, 134.3, 140.4, 143.5, 148.5, 155.9, 161.5, 163.5, and 170.4; *m*/*z* (ES +ve mode) 487 (MNa^+^, base peak).

#### (*S*)-{2-[(5-Nitrothiazol-2-yl)carbamoyl]phenyl}-2-amino-3-methylbutanoate, Hydrochloride **18**

As described for **5a**,^13^ the preceding Boc derivative **16** (0.250 g, 0.54 mmol) was suspended in CH_2_Cl_2_ (5 mL) and 4 M HCl in dioxane (2 mL) was added with stirring at 20 °C. A solution resulted after a few minutes, but the solid soon began to precipitate. After 16 h, the reaction was diluted with ether, briefly stirred, and then cooled to 0 °C to complete precipitation; filtration afforded the title compound **18** (0.205 g, 93%) as a light-yellow solid. Found: *m*/*z*, 387.07222. C_15_H_16_N_4_O_5_SNa (MNa^+^) requires *m*/*z*, 387.0739; δ_H_ [(CD_3_)_2_SO] 1.05 (6H, 2d, *Me*_2_CH), 2.37 (1H, m, Me_2_*CH*CH), 4.17 (1H, m, CH*CH*NH), 7.49 (1H, d, ArH), 7.53 (1H, t, ArH), 7.76 (1H, t, ArH), 7.90 (1H, d, ArH), 8.74 (1H, s, thiazole 4-H), 8.80–8.90 (3H, br s, NH_3_^+^), and 13.80 (1H, br s, CONH); δ_C_ 18.3, 18.4, 29.6, 58.0, 123.8, 126.3, 127.2, 130.3, 134.0, 142.6, 143.0, 147.9, 162.3, 165.6, and 167.9; *m*/*z* 387 (ES +ve mode, MNa^+^ for free amine, 100%).

#### (*S*)-{2-[(5-Chloro-1,3-thiazol-2-yl)carbamoyl]phenyl}-2-amino-3-methylbutanoate, Hydrochloride **19**

Boc-Val-OH (0.65 g, 3.00 mmol) and RM4848 **4** (0.51 g, 2.00 mmol) in THF (7.5 mL) were coupled using EDC (0.57 g, 3.00 mmol) and DMAP (0.37 g, 3.00 mmol) at 20 °C as described for the preparation of **16**. After 20 h, the mixture was filtered through Celite and worked up for a neutral product. Chromatography using a gradient of 25–40% EtOAc/hexane removed unreacted **4**. Product fractions were pooled and evaporated to afford the crude product **17** (0.487 g, 54%), which was immediately progressed. This material (0.47 g, 1.04 mmol) in CH_2_Cl_2_ (5 mL) was treated with 4 M HCl in dioxane (3 mL) at 0 °C. Further 4 M HCl in dioxane (2 mL) was added after 2.25 h; after 6 h, the mixture was stored at 0 °C overnight. Isolation of the product was completed as described for **18** to afford the title HCl salt **19** (0.317 g, 78%); δ_H_ [(CD_3_)_2_SO] 1.05 (6H, 2d, *Me*_2_CH), 2.36 (1H, m, Me_2_*CH*CH), 4.09 (1H, m, CH*CH*NH), 7.44–7.53 (2H, m, ArH), 7.60 (1H, s, thiazole 4-H), 7.69 (1H, t, ArH), 7.89 (1H, d, ArH), 8.85 (3H, br s, NH_3_^+^), and 12.50 (1H, br s, NH). This product was immediately progressed by rearrangement: see below.

#### (*S*)-*N*-(3,3-Dimethyl-1-((5-nitrothiazol-2-yl)amino)-1-oxobutan-2-yl)-2-hydroxybenzamide **8**

A suspension of prodrug ester **5a** (0.100 g, 0.24 mmol)^13^ in 1,4-dioxan (3 mL) was stirred at 20 °C and treated with triethylamine (0.04 mL, 0.29 mmol). After 0.5 h, the mixture was diluted with EtOAc (20 mL) and THF (5 mL, to improve solubility), washed with 7% aq. citric acid solution and brine, dried, and then evaporated to obtain a pale-yellow solid (0.085 g), which was chromatographed, applying in CH_2_Cl_2_ and eluting with EtOAc/hexane, 1:3. Appropriate fractions were pooled and evaporated to give the product **8** (0.047 g, 52%), which was recrystallized from EtOAc/hexane to give an analytical sample. Found: C, 50.8; H, 4.8; N, 14.8; S, 8.5; *m*/*z*, 401.0887; C_16_H_18_N_4_O_5_S requires C, 50.8; H, 4.8; N, 14.8; S, 8.5%; C_16_H_18_N_4_O_5_SNa requires *m*/*z*, 401.0896; δ_H_ [(CD_3_)_2_SO] 1.06 (9H, s, Me_3_C), 4.73 (1H, d, *J* = 7.6 Hz, *CH*NH), 6.94 (1H, t, ArH), 6.99 (1H, d, ArH), 7.40 (1H, t, ArH), 7.92 (1H, dd, ArH), 8.66 (1H, s, thiazole 4-H), 8.97 (1H, d, *J* = 7.6 Hz, CH*NH*), 11.55 (1H, br s), and 13.43 (1H, br s); δ_C_ 26.8, 34.6, 61.1, 117.3, 118.3, 120.0, 131.0, 133.8, 142.5, 143.1, 157.1, 161.4, 166.0, and 171.9.

#### (*S*)-*N*-(3,3-Dimethyl-1-((5-chlorothiazol-2-yl)amino)-1-oxobutan-2-yl)-2-hydroxybenzamide **20**

This was prepared similarly to **8** from RM5064 **6** (0.404 g, 1.00 mmol),^13^ affording the desired product **20** (0.279 g, 76%) as a white solid. Found: C, 52.2; H, 4.9; N, 11.5; S, 8.4; *m*/*z*, 390.0647. C_16_H_18_ClN_3_O_3_S requires C, 52.2; H, 4.9; N, 11.4; S, 8.7%; C_16_H_18_^35^ClN_3_O_3_S.Na (MNa^+^) requires *m*/*z*, 390.0655; δ_H_ [(CD_3_)_2_SO] 1.06 (9H, s, Me_3_C), 4.85 (1H, d, *J* = 9.2 Hz, *CH*NH), 6.90 (1H, t, ArH), 6.99 (1H, d, ArH), 7.09 (1H, br d, CH*NH*), 7.43 (1H, t, ArH), 7.49 (1H, d, ArH), 7.72 (1H, s, thiazole 4-H), 11.87 and 12.36 (2H, 2 br s, NH and OH); δ_C_ 26.5, 35.6, 59.9, 113.8, 118.7, 119.0, 122.0, 125.7, 134.6, 134.8, 156.4, 161.5, 169.1, and 169.8.

#### (*S*)-2-Hydroxy-*N*-(3-methyl-1-((5-nitrothiazol-2-yl)amino)-1-oxobutan-2-yl)benzamide **21**

This was prepared similarly to **8** from the prodrug ester **18**(13) (0.041 g, 0.102 mmol), affording the desired product **22** (0.025 g, 67%) as a pale-brown solid after chromatography. Found: *m*/*z*, 387.0728; C_15_H_16_N_4_O_5_S requires *m*/*z*, 387.0739; δ_H_ [CDCl_3_] 1.15 (6H, t, *Me*_2_CH), 2.45 (1H, m, Me_2_*CH*CH), 4.74 (1H, t, CH*CH*NH), 6.90 (1H, t, ArH), 6.98 (1H, d, ArH), 7.27 (1H, d, NH), 7.44 (1H, t, ArH), 7.54 (1H, d, ArH), 8.40 (1H, s, thiazole 4-H), and 11.38 (1H, br s, NH); δ_C_ 18.6, 19.4, 30.6, 59.2, 113.6, 118.6, 119.4, 126.2, 135.2, 140.3, 144.0, 160.4, 160.9, 170.4, and 170.8;

#### (*S*)-2-Hydroxy-*N*-(3-methyl-1-((5-chlorothiazol-2-yl)amino)-1-oxobutan-2-yl)benzamide **22**

This was prepared similarly to **8** from the prodrug ester **19** (0.300 g, 0.77 mmol), affording the desired product **22** (0.153 g, 56%) as a white solid. Found: C, 50.8; H, 4.6; N, 11.8; S, 8.8; *m*/*z*, 376.0490. C_15_H_16_ClN_3_O_3_S requires C, 50.9; H, 4.6; N, 11.9; S, 9.06%; C_15_H_16_^35^ClN_3_O_3_S.Na (MNa^+^) requires *m*/*z*, 376.0499; δ_H_ [(CD_3_)_2_SO] 0.95-0.98 (6H, 2d, *Me*_2_CH), 2.22 (1H, m, Me_2_*CH*CH), 4.63 (1H, t, *J* = 7.6 Hz, CH*CH*NH), 6.92–6.97 (2H, m, ArH), 7.41 (1H, t, ArH), 7.55 (1H, s, thiazole 4-H), 7.98 (1H, d, ArH), 8.90 (1H, d, *J* = 7.6 Hz, NH), 11.77 (1H, s), and 12.68 (1H, s); δ_C_ 18.9, 19.6, 30.7, 58.7, 117.2, 117.5, 118.8, 119.6, 130.1, 134.0, 136.2, 155.9, 158.4, 167.6, and 171.2.

#### (*S*)-3,3-Dimethyl-1-(2-((5-nitrothiazol-2-yl)carbamoyl)phenoxy)-1-oxobutan-2-aminium 4-methylbenzenesulfonate **5b**

*p*-Toluenesulfonic acid (15.2 g, 20.0 mmol) was added in portions to a solution of 2-((5-nitrothiazol-2-yl)carbamoyl) phenyl (*S*)-2-((*tert*-butoxycarbonyl)amino)-3,3-dimethyl butanoate (4.0 g, 8.4 mmol) **14** in ethyl acetate (80 mL), and the reaction was stirred overnight. A white precipitate began to form within ca. 2 h. The reaction was cooled to 0 °C; then, the precipitate was filtered off, washed with diethyl ether, and dried in vacuo to afford a white crystalline solid, which was recrystallized from hot ethanol to afford the title compound **5b** as a pale-yellow crystalline solid (3.07 g, 66% yield). Found: C, 49.61; H, 4.86; N, 10.13; S, 11.39; *m*/*z* 379.1073. C_23_H_26_N_4_O_8_S_2_ requires: C, 50.17; H, 4.76; N, 10.18; S, 11.65; C_16_H_18_N_4_O_5_S [M + H]^+^ requires *m*/*z*, 379.1071; δ_H_ [(CD_3_)_2_SO]1.11 (9H, s), 2.30 (3H, s), 4.10 (1 H, s), 7.12 (2H, d, *J* = 7.6 Hz), 7.41 (1H, d, *J* = 8.4 Hz), 7.49–7.55 (3H, m), 7.74 (1H, t, *J* = 7.6 Hz), 7.86 (1H, d, *J* = 7.6 Hz), 8.47 (3H, br s), 8.72 (1H, s), and 13.78 (1H, br s); δ_C_ 21.2, 26.5, 34.0, 61.6, 123.6, 126.0, 126.6, 127.3, 128.6, 130.2, 133.9, 138.3, 142.6, 143.0, 147.7, 162.4, 165.9, and 167.6; *m*/*z* 379 (ES +ve mode, MH^+^ for amine).

#### (*S*)-3,3-Dimethyl-1-(2-((5-nitrothiazol-2-yl)carbamoyl)phenoxy)-1-oxobutan-2-aminium Methanesulfonate **5c**

Methanesulfonic acid (0.33 mL, 5.08 mmol) was added dropwise at 20 °C to a solution of 2-((5-nitrothiazol-2-yl)carbamoyl) phenyl (*S*)-2-((*tert*-butoxycarbonyl)amino)-3,3-dimethyl butanoate **14** (1.04 g, 2.17 mmol) in ethyl acetate (5 mL), and the reaction was stirred overnight. The resulting white precipitate was then filtered off, washed with diethyl ether, and dried in vacuo. The crude product was recrystallized from hot ethanol to afford the title compound **5c** as a white crystalline solid (0.37 g, 37% yield). Found: C, 43.02; H, 4.73; N, 11.79; S, 13.56; *m*/*z* 379.1078. C_17_H_22_N_4_O_8_S_2_ requires C, 43.03; H, 4.67; N, 11.81; S, 13.51; C_16_H_18_N_4_O_5_S [M + H]^+^ requires *m*/*z*, 379.1071; ^1^H NMR (400 MHz, (CD_3_)_2_SO) δ_H_ 1.11 (9H, s), 2.34 (3H, s), 4.08 (1H, s), 7.44 (1H, d, *J* = 8.1 Hz), 7.52 (1H, t, *J* = 7.6 Hz), 7.75 (1H, td, *J*_1_ = 7.5 Hz, *J*_2_ = 1.5 Hz), 7.86 (1H, dd, *J*_1_ = 7.7 Hz, *J*_2_ = 1.4 Hz), 8.48 (3H, br s), 8.71 (1H, s), and 13.77 (1H, br s). ^13^C NMR (100 MHz, (CD_3_)_2_SO) δ_C_ 26.53, 33.94, 61.61, 123.69, 126.57, 127.24, 130.19, 133.85, 142.63, 142.99, 147.72, 162.32, 165.83, and 167.62; *m*/*z* 379 (ES +ve mode, MH^+^ for amine).

## Antiviral Assay

### Cell Culture and Treatments

Madin–Darby canine kidney (MDCK) cells (American Type Culture Collection, ATCC) were grown at 37 °C in a 5% CO_2_ atmosphere in an RPMI-1640 medium (LONZA-CAMBREX, Basel, Switzerland), supplemented with 10% fetal calf serum (FCS), 2 mM glutamine, and antibiotics. Tizoxanide (Romark Laboratories, L.C.) and derived peptides, dissolved in a dimethyl sulfoxide (DMSO) stock solution, were diluted in culture medium, added to infected cells after a one-hour virus adsorption period, and maintained in the medium for the duration of the experiment. Controls received equal amounts of the DMSO vehicle, which did not affect cell viability or virus replication.

Cell viability was determined by the 3-(4,5-dimethylthiazol-2-yl)-2,5-diphenyltetrazolium bromide (MTT) to MTT formazan conversion assay (Sigma-Aldrich), as described.^28,29^ The 50% cytotoxic dose (CC_50_) was calculated using Prism 5.0 software (Graph-Pad Software Inc.).^28^

### Virus Preparation, Infection, and Titration

The influenza A virus (IAV) strain A/PuertoRico/8/1934(H1N1) (PR8), a prototype strain of the H1N1 IAV subtype, was utilized in this study. PR8 virus was grown in the allantoic cavity of 10 day old embryonated eggs; the virus titer was determined by a plaque assay, as described previously.^30,31^

Confluent cell monolayers were mock-infected or infected with PR8 virus for 1 h at 37 °C at a multiplicity of infection (MOI) of 5 HAU (hemagglutinating units)/10^5^ cells, as described.^30,31^ After the adsorption period, the viral inoculum was removed and cells were treated with different concentrations (0.1, 1, 5, 10, and 50 μg/mL) of each compound or vehicle and maintained at 37 °C in an RPMI-1640 medium containing 2% FCS for 24 h; in parallel, cell viability was determined in mock-infected cells by the MTT assay, as described above. Virus yield was determined 24 h post infection (p.i.) by HA titration, as described.^31^ Compounds’ IC_50_ (50% inhibitory concentration) and IC_90_ (90% inhibitory concentration) were calculated using Prism 5.0 software.

## Supporting Information Available

Photocopy ^1^H and ^13^C NMR spectra for compounds **8**, **20**, **21**, and **22**; full time-course NMR spectra for the degradation of **5a**; proposed mechanism for the rearrangement of **5a** to **8**; and analytical HPLC traces for compounds **8**, **20**, **21**, and **22** (PDF)

## References

[ref1] Rossignol J.-F. Nitazoxanide, a first-in-class broad-spectrum antiviral agent. Antiviral Res. 2014, 110, 94–103. 10.1016/j.antiviral.2014.07.014. 25108173 PMC7113776

[ref2] Korba B. E.; Montero A. B.; Farrar K.; Gaye K.; Mukerjee S.; Ayers M. S.; Rossignol J.-F. Nitazoxanide, tizoxanide and other thiazolides are potent inhibitors of hepatitis B virus and hepatitis C virus replication. Antiviral Res. 2008, 77, 56–63. 10.1016/j.antiviral.2007.08.005. 17888524

[ref3] Stachulski A. V.; Pidathala C.; Row E.; Sharma R.; Berry N. G.; Iqbal M.; Bentley J.; Allman S. A.; Edwards G. E.; Helm A.; Hellier J.; Korba B. E.; Semple J. E.; Rossignol J.-F. Thiazolides as novel antiviral agents. 1. Inhibition of hepatitis B virus replication. J. Med. Chem. 2011, 54, 4119–4132. 10.1021/jm200153p. 21553812 PMC3124649

[ref4] Stachulski A. V.; Pidathala C.; Row E.; Sharma R.; Berry N. G.; Lawrenson A. S.; Moores S. L.; Iqbal M.; Bentley J.; Allman S. A.; Edwards G. E.; Helm A.; Hellier J.; Korba B. E.; Semple J. E.; Rossignol J.-F. Thiazolides as novel antiviral agents. 2. Inhibition of hepatitis C virus replication. J. Med. Chem. 2011, 54, 8670–8680. 10.1021/jm201264t. 22059983

[ref5] Stachulski A. V.; Santoro M. G.; Piacentini S.; Belardo G.; La Frazia S.; Pidathala C.; Row E. C.; Berry N. G.; Iqbal M.; Allman S. A.; Semple J. E.; Eklov B. M.; O’Neill P. M.; Rossignol J.-F. Second-generation nitazoxanide derivatives: thiazolides are effective inhibitors of the influenza A virus. Future Med. Chem. 2018, 10, 851–862. 10.4155/fmc-2017-0217. 29629834

[ref6] Rossignol J. −F.; Abu-Zekry M.; Abeer H.; Santoro M. G. Effect of nitazoxanide in treating severe rotavirus diarrhea: a randomized, double-blind, placebo-controlled trial. Lancet 2006, 368, 124–129. 10.1016/S0140-6736(06)68852-1. 16829296

[ref7] Haffizulla J.; Hartmann A.; Hoppers M.; Resnick H.; Samudrala S.; Ginocchio C.; Bardin M.; Rossignol J-F. Effect of nitazoxanide in adults and adolescents with acute uncomplicated influenza: a double-blind, randomized, placebo-controlled, Phase IIb/III trial. Lancet Infect. Dis. 2014, 14, 609–618. 10.1016/S1473-3099(14)70717-0. 24852376 PMC7164783

[ref8] NIH trial: NCT04486313. https://clinicaltrials.gov/ct2/show/NCT04486313, July 24, 2020.

[ref9] NIH trial: NCT04406246. https://clinicaltrials.gov/ct2/show/NCT04406246, March 29, 2021.

[ref10] Rautio J.; Kumpulainen H.; Heimbach T.; Oliyai R.; Oh D.; Jarvinen T.; Savolainen J. Prodrugs: design and clinical applications. Nat. Rev. Drug Discovery 2008, 7, 255–270. 10.1038/nrd2468. 18219308

[ref11] Rautio J.; Meanwell N. A.; Di L.; Hageman M. J. The expanding role of prodrugs in contemporary drug design and development. Nat. Rev. Drug Discovery 2018, 17, 559–587. 10.1038/nrd.2018.46. 29700501

[ref12] Beauchamp L. M.; Orr G. F.; Demiranda P.; Burnette T.; Krenitsky T. A. Amino-acid ester prodrugs of acyclovir. Antiviral Chem. Chemother. 1992, 3, 157–164. 10.1177/095632029200300305.

[ref13] Stachulski A. V.; Swift K.; Cooper M.; Reynolds S.; Norton D.; Slonecker S. D.; Rossignol J. −F. Synthesis and pre-clinical studies of new amino-acid ester thiazolide prodrugs. Eur. J. Med. Chem. 2017, 126, 154–159. 10.1016/j.ejmech.2016.09.080. 27750149 PMC7125651

[ref14] Galande A. K.; Bramlett K. S.; Trent J. O.; Burris T. P.; Wittliff J. L.; Spatola A. F. Potent inhibitors of LXXLL-based protein-protein interactions. ChemBioChem 2005, 6, 1991–1998. 10.1002/cbic.200500083. 16222726

[ref15] Stachulski A. V.; Rossignol J.-F.; Pate S.; Taujanskas J.; Robertson C. M.; Aerts R.; Pascal E.; Piacentini S.; La Frazia S.; Santoro M. G.; O’Neill P. M. Synthesis, antiviral activity, preliminary pharmacokinetics and structural parameters of thiazolide amine salts. Future Med. Chem. 2021, 13, 1731–1741. 10.4155/fmc-2021-0055. 34402654

[ref16] Wuts P. G. M.; Greene T. W. Greene’s Protective Groups in Organic Synthesis, 4th ed.; Wiley-Interscience, 2007; p 729.

[ref17] Granero G. E.; Amidon G. L. Stability of valacyclovir: implications for its oral bioavailability. Int. J. Pharm. 2006, 317, 14–18. 10.1016/j.ijpharm.2006.01.050. 16759825

[ref18] Imramovský A.; Vinsova J.; Ferriz J. M.; Kunes J.; Pour M.; Dolezal M. Salicylanilide esterification: unexpected formation of novel seven-membered rings. Tetrahedron Lett. 2006, 47, 5007–5011. 10.1016/j.tetlet.2006.05.110.

[ref19] Vinšová J.; Imramovský A.; Kratky M.; Ferriz J. M.; Palat K.; Lycka A.; Ruzicka A. An unprecedented rearrangement of salicylanilide derivatives: imidazolinone intermediate formation. Tetrahedron Lett. 2010, 51, 23–26. 10.1016/j.tetlet.2009.10.084.

[ref20] Imramovský A.; Vinsova J.; Ferriz J. M.; Buchta V.; Jampilek J. Salicylanilide esters of N-protected amino acids as novel antimicrobial agents. Bioorg. Med. Chem. Lett. 2009, 19, 348–351. 10.1016/j.bmcl.2008.11.080. 19081718

[ref21] Xu J.; Berastegui-Cabrera J.; Chen H.; Pachon J.; Zhou J.; Sanchez-Cespedes J. Structure-activity relationship studies on diversified salicylamide derivatives as potent inhibitors of human adenovirus infection. J. Med. Chem. 2020, 63, 3142–3160. 10.1021/acs.jmedchem.9b01950. 32045239

[ref22] Jurgeit A.; McDowell R.; Moese S.; Meldrum E.; Schwendener R.; Greber U. F. Niclosamide is a proton carrier and targets acidic endosomes with broad antiviral effects. PloS Pathog. 2012, 8, e1002976 10.1371/journal.ppat.1002976. 23133371 PMC3486884

[ref23] Wu C. J.; Jan J. T.; Chen C. M.; Hsieh H. P.; Hwang D. R.; Liu H. W.; Liu C. Y.; Huang H. W.; Chen S. C.; Hong C. F.; Liu R. K.; Chao Y. S.; Hsu J. T. Inhibition of severe acute respiratory syndrome coronavirus replication by niclosamide. Antimicrob. Agents Chemother. 2004, 48, 2693–2696. 10.1128/AAC.48.7.2693-2696.2004. 15215127 PMC434198

[ref24] Brenner M.; Zimmermann J. P.; Wehrmuller J.; Quitt P.; Photaki I. Eine neue umlagerungsreaktion und ein neues prinzip zum aufbau von peptidketten. Experientia 1955, 11, 397–399. 10.1007/BF02158504.

[ref25] Brenner M.; Zimmermann J. P.; Wehrmüller J.; Quitt P.; Hartmann A.; Schneider W.; Beglinger U. Aminoacyl-einlagerung. 1. Mitteilung. Definition, übersicht und beziehung zur peptidsynthese. Helv. Chim. Acta 1957, 40, 1497–1517. 10.1002/hlca.19570400533.

[ref26] Titherley A. W.; Hicks W. L. Labile isomerism among benzoyl derivatives of salicylamide. J. Chem. Soc. 1905, 87, 1207–1229. 10.1039/CT9058701207.

[ref27] Rossignol J.-F.; Stachulski A. V. Syntheses and antibacterial activities of tizoxanide, an *N*-(nitrothiazolyl)salicylamide, and its *O*-aryl glucuronide. J. Chem. Res. (S), 1999, 1, 44–45. 10.1177/174751989902300128.

[ref28] Piacentini S.; La Frazia S.; Riccio A.; Pedersen J. Z.; Topai A.; Nicolotti O.; Rossignol J.-F.; Santoro M. G. Nitazoxanide inhibits paramyxovirus replication by targeting the fusion protein folding: role of glycoprotein-specific thiol oxidoreductase ERp57. Sci. Rep. 2018, 8, 10425 10.1038/s41598-018-28172-9. 29992955 PMC6041319

[ref29] Santoro M. G.; Amici C.; Elia G.; Benedetto A.; Garaci E. Inhibition of virus protein glycosylation as the mechanism of the antiviral action of prostaglandin-A in sendai-virus infected cells. J. Gen. Virol. 1989, 70, 789–800. 10.1099/0022-1317-70-4-789. 2543761

[ref30] La Frazia S.; Piacentini S.; Riccio A.; Rossignol J. F.; Santoro M. G. The second generation thiazolide haloxanide is a potent inhibitor of avian influenza virus replication. Antiviral Res. 2018, 157, 159–168. 10.1016/j.antiviral.2018.06.008. 29908209

[ref31] Belardo G.; Cenciarelli O.; La Frazia S.; Rossignol J. F.; Santoro M. G. Synergistic effect of nitazoxanide with neuraminidase inhibitors against influenza A viruses in vitro. Antimicrob. Agents Chemother. 2015, 59, 1061–1069. 10.1128/AAC.03947-14. 25451059 PMC4335909

