# Supplementary material for: Thiazolide Prodrug Esters and Derived Peptides: Synthesis and Activity
Source: ACS Bio Med Chem Au. 2023 Apr 11;3(4):327–34. doi: 10.1021/acsbiomedchemau.2c00083 (PMC10436260; doi:10.1021/acsbiomedchemau.2c00083)
Supplement: Supplementary file 1 — bg2c00083_si_001.pdf [file bg2c00083_si_001.pdf]

Thiazolide Prodrug Esters and Derived Peptides: Synthesis and Activity

Andrew V. Stachulski, *[a] Jean-Francois Rossignol,[b] Sophie Pate,[a] Joshua Taujanskas,[a] Jonathan A. Iggo,[a] Rudi Aerts,[c] Etienne Pascal,[c] Sara Piacentini,[d] Simone La Frazia,[d] M. Gabriella Santoro,[d,e] Lieven van Vooren,[f] Liesje Sintubin,[f] Mark Cooper,[g] Karl Swift[g] and Paul M. O'Neill[a]

[a] Donnan and Robert Robinson Laboratories, Department of Chemistry, University of Liverpool, Liverpool L69 7ZD, UK

[b] Romark Laboratories, L.C., Tampa, 33609 FL, United States

[c] Romark Belgium BVBA, Roosveld 6, 3400 Landen, Belgium

[d] Department of Biology, University of Rome Tor Vergata, 00133 Rome, Italy

[e] Institute of Translational Pharmacology, CNR, Area della Ricerca di Roma 2, Via Fosso del Cavaliere 00133 Roma, Italy

[f] Ardena Gent NV, Kleimoer 4, 9030 Mariakerke, Belgium

[gß] Bio-Techne, Avonmouth, Bristol BS11 9QD, UK

Supporting Information

1) Photocopy 1H and 13C NMR spectra for compounds **8**, **20**, **21** and **22**

2) Full time course NMR spectra for the degradation of **5a**

3) Proposed mechanism for the rearrangement of **5a** to **8**

4) Analytical HPLC traces for compounds **8**, **20**, **21 and 22**.

[a] To whom correspondence should be addressed: stachuls@liv.ac.uk, tel. 0151-794-3482

1) Photocopy NMR spectra for compounds **8**, **20**, **21** and **22**

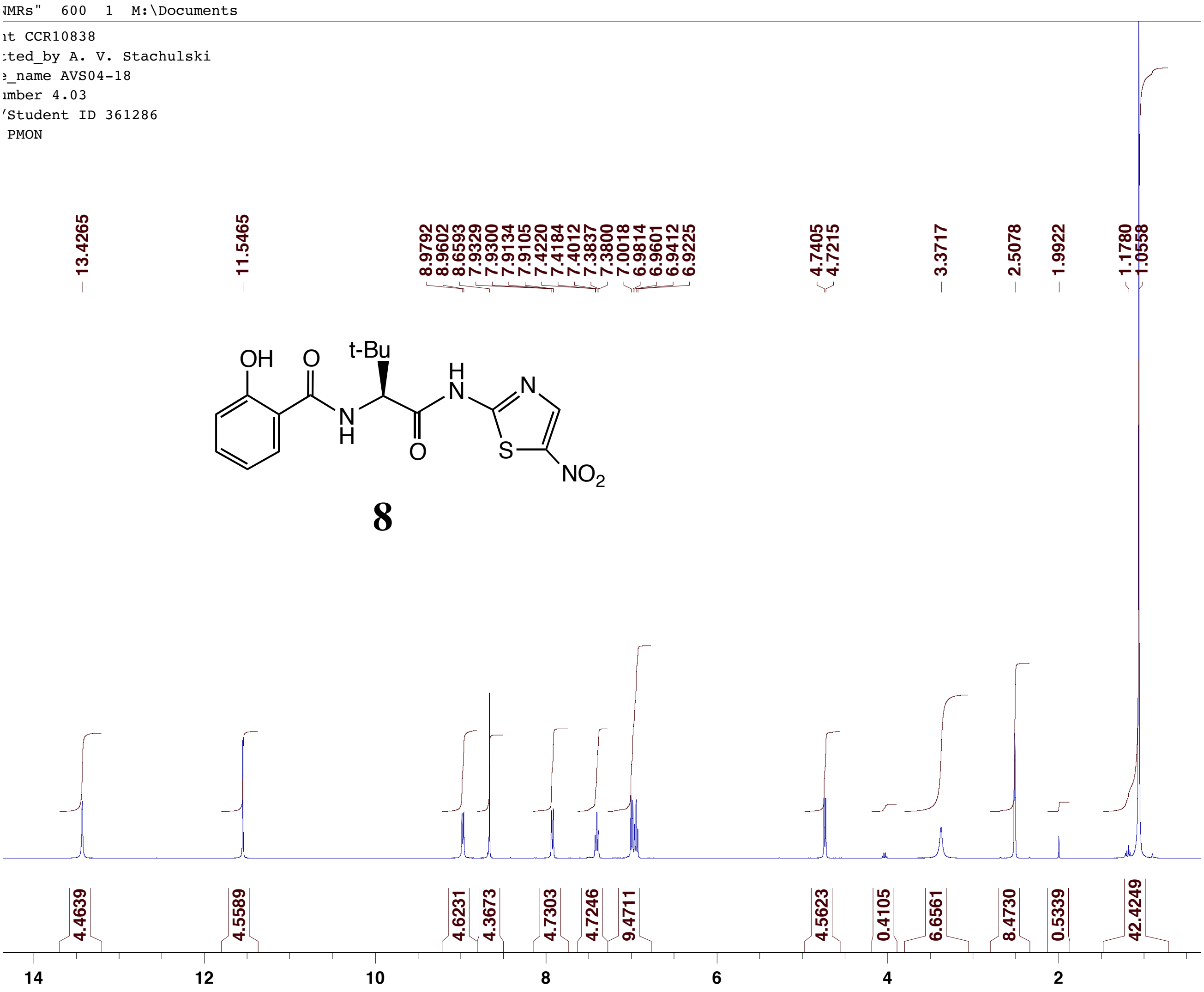

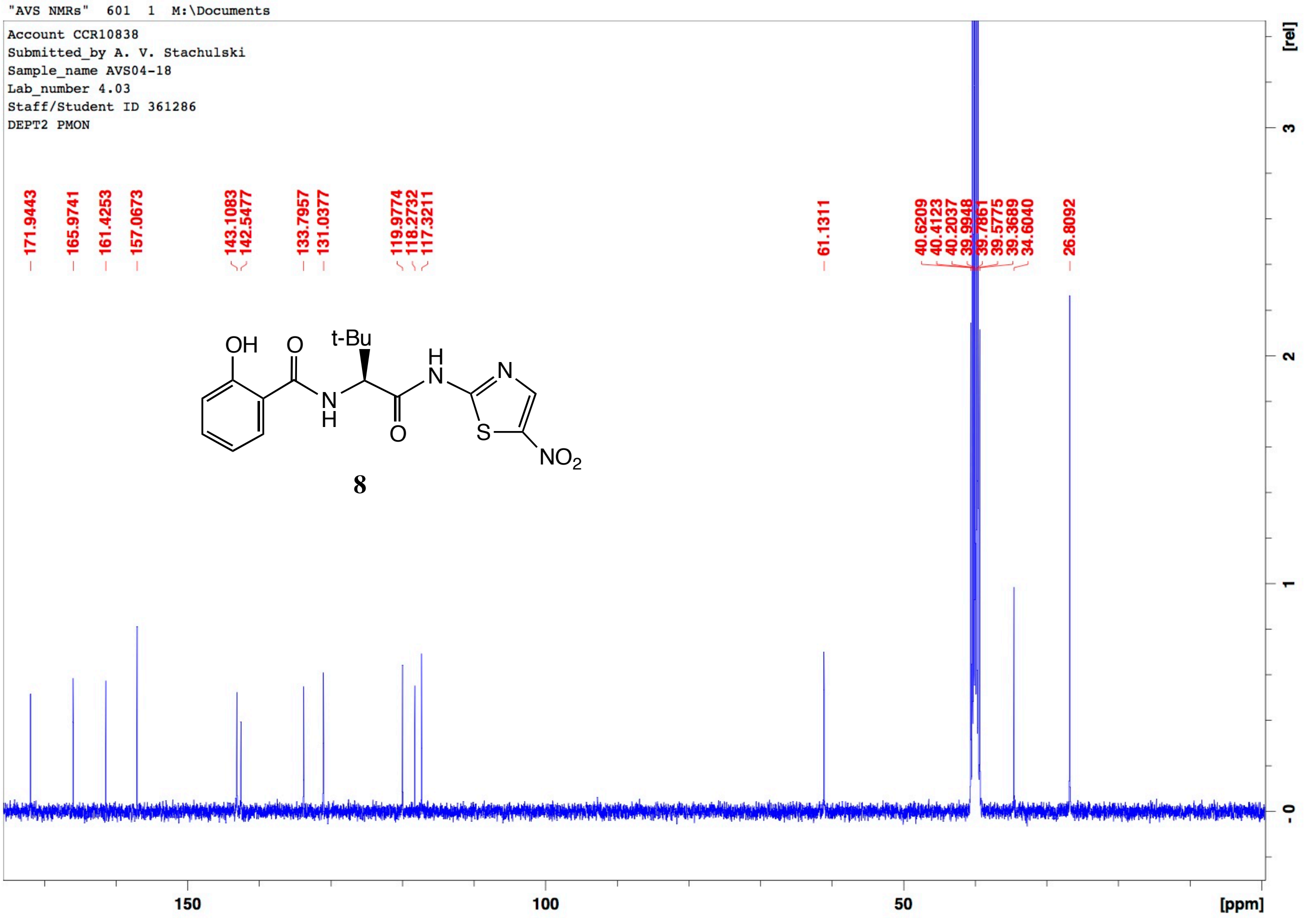

"AVS NMRs" 601 1 M:\Documents
Account CCR10838
Submitted_by A. V. Stachulski
Sample_name AVS04-18
Lab_number 4.03
Staff/Student ID 361286
DEPT2 PMON
171.9443
165.9741
161.4253
157.0673
143.1083
142.5477
133.7957
131.0377
119.9774
118.2732
117.3211
61.1311
40.6209
40.4123
40.2037
39.9948
39.7861
39.5775
39.3689
34.6040
26.8092
OH
O
t-Bu
H
N
N
H
O
N
S
NO2
8
[rel]
3
2
1
0
150
100
50
[ppm]

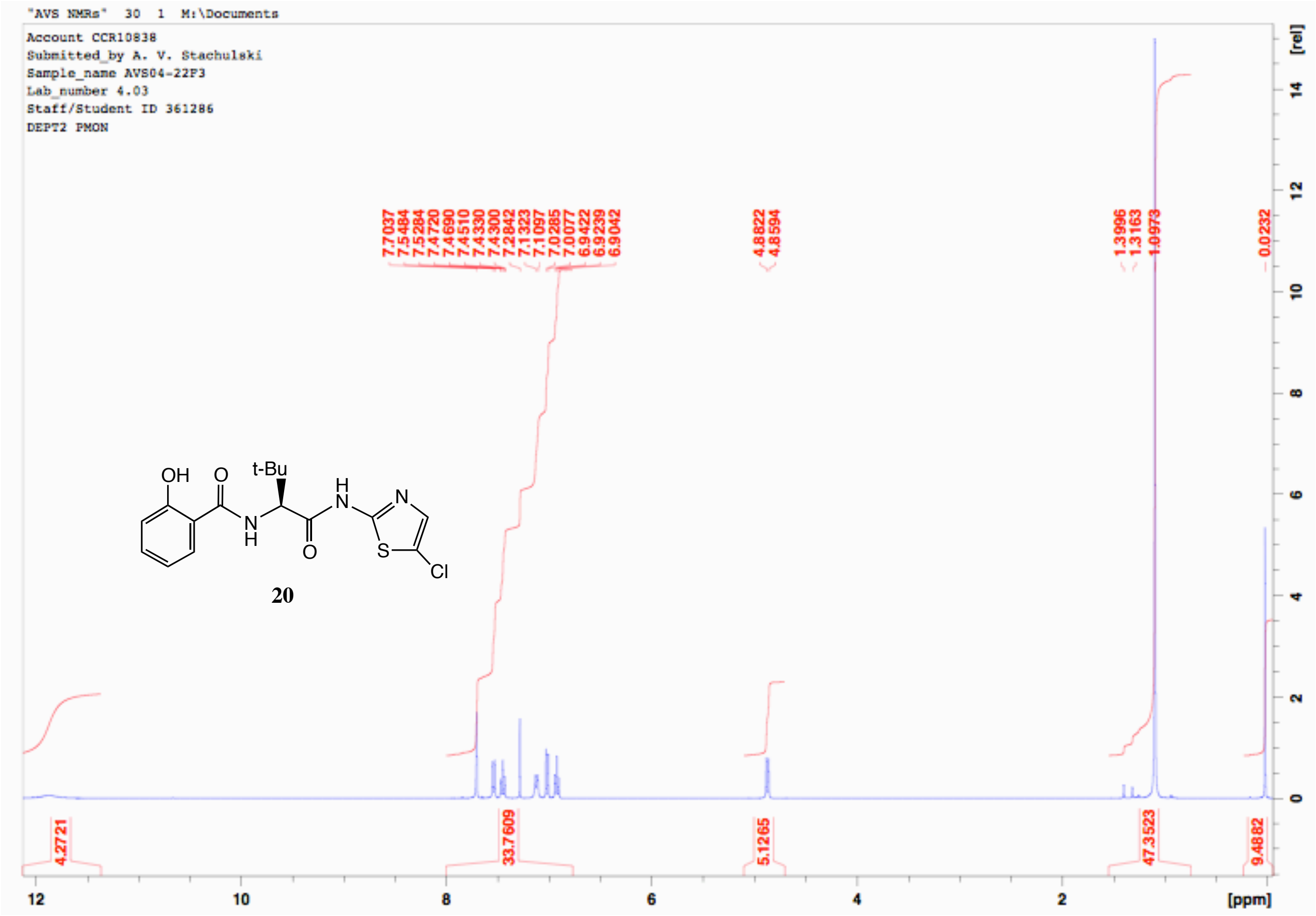
"AVS NMRs" 30 1 M:\Documents
Account CCR10838
Submitted_by A. V. Stachulski
Sample_name AVS04-22P3
Lab_number 4.03
Staff/Student ID 361286
DEPT2 PMON
7.7037 7.5484 7.5284 7.4720 7.4690 7.4510 7.4330 7.4300 7.2842 7.1323 7.1097 7.0285 7.0077 6.9422 6.9239 6.9042
4.8822 4.8594
1.3996 1.3163 1.0973
-0.0232
4.2721
33.7609
5.1265
47.3523
9.4882
[ppm]
[rel]
OH O t-Bu H N N S Cl O H

20

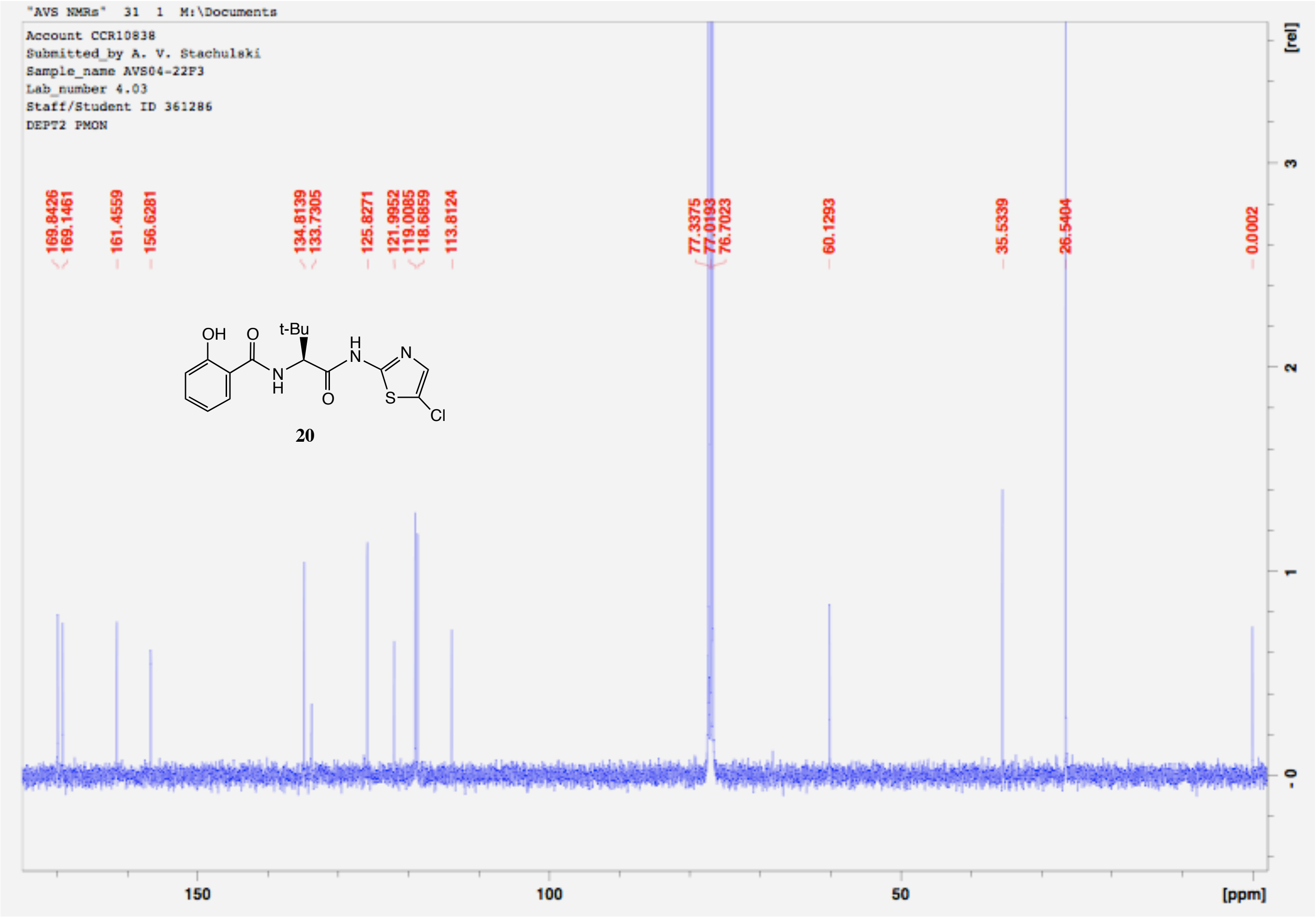
"AVS NMRs" 31 1 M:\Documents
Account CCR10838
Submitted_by A. V. Stachulski
Sample_name AVS04-22F3
Lab_number 4.03
Staff/Student ID 361286
DEPT2 PMON
169.8426
169.1461
161.4559
156.6281
134.8139
133.7305
125.8271
121.9952
119.0085
118.6859
113.8124
77.3375
77.0193
76.7023
60.1293
35.5339
26.5404
0.0002
OH
O
t-Bu
H
N
N
H
O
N
S
Cl
20
150
100
50
[ppm]
[rel]

"AVS NMRs" 490 1 M:\Documents

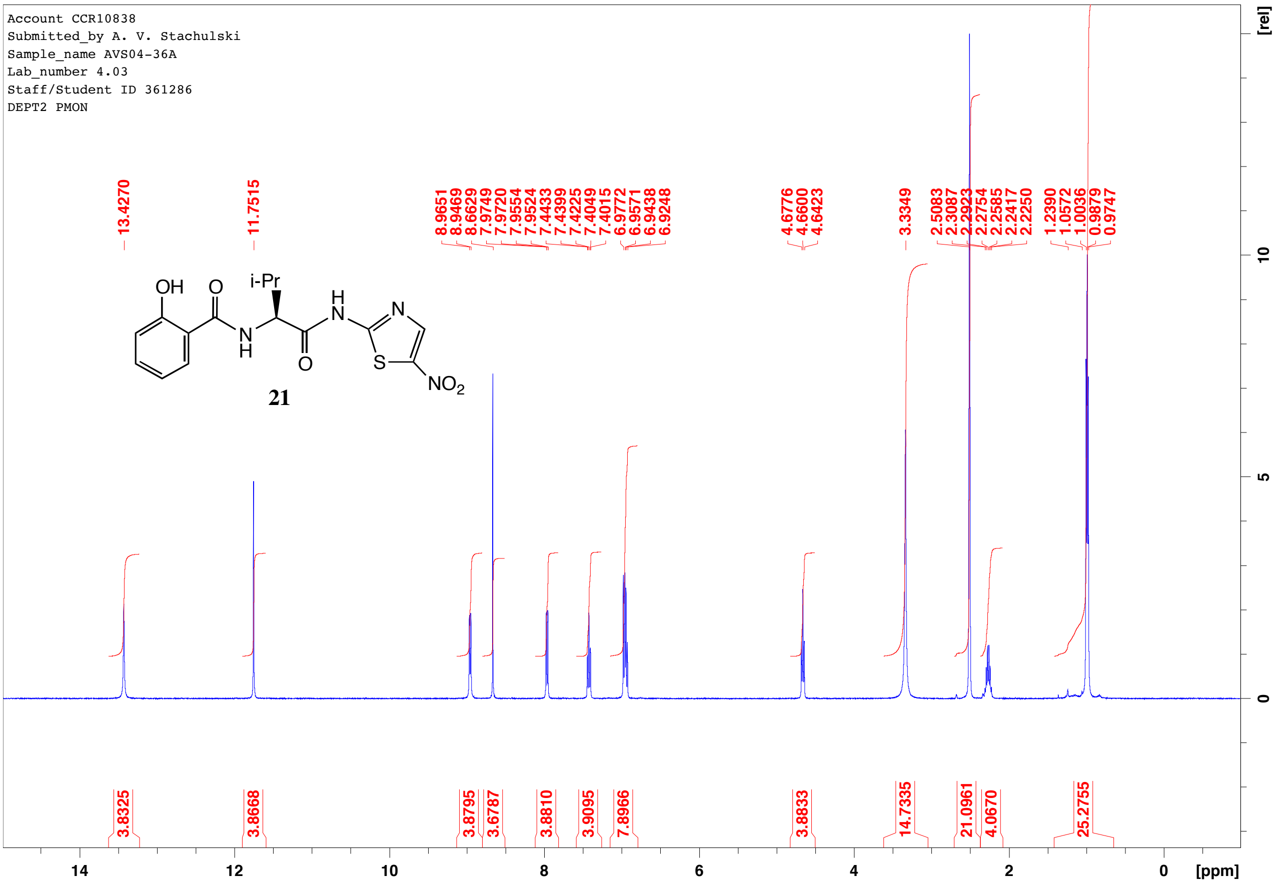

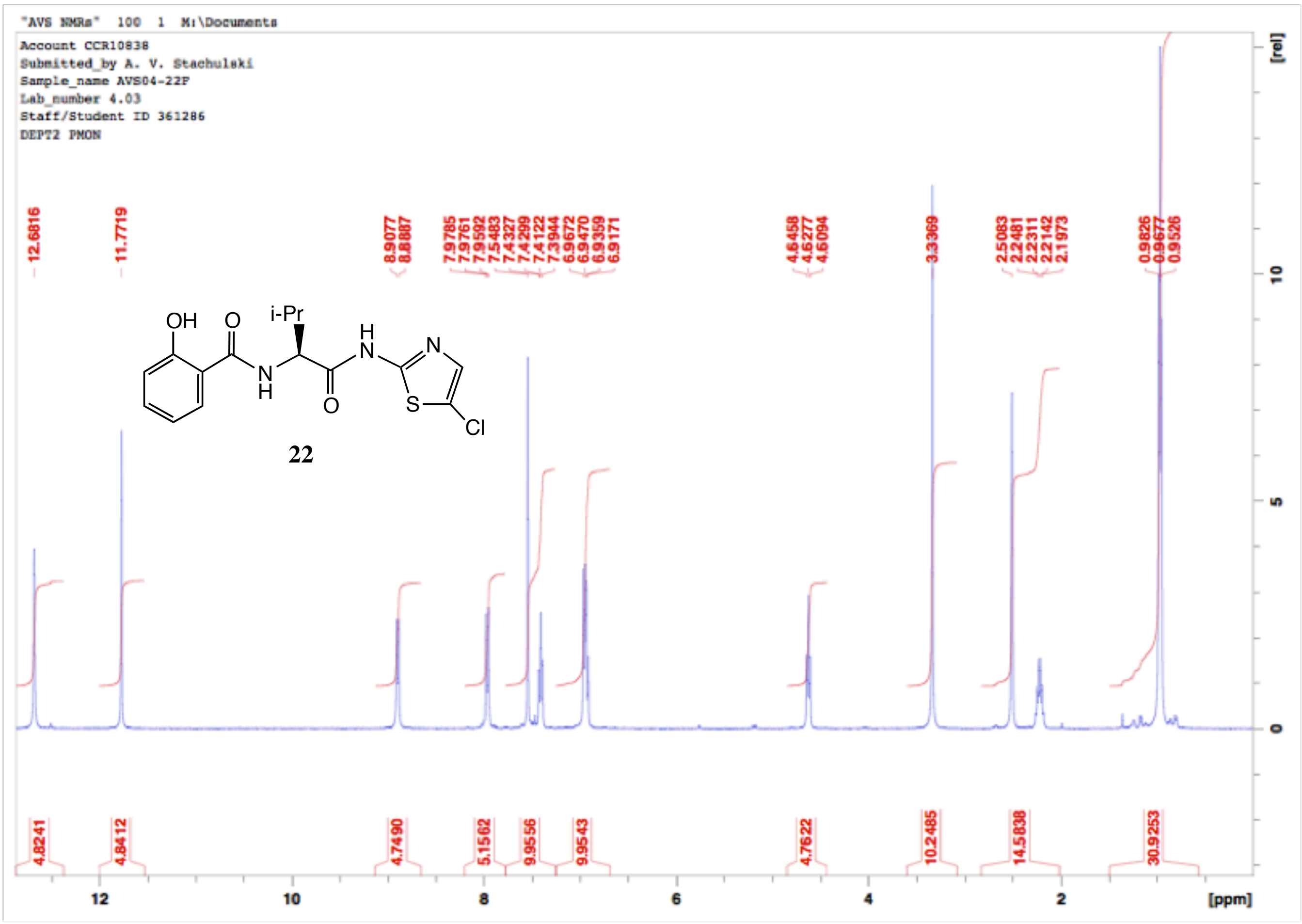
"AVS NMRs" 100 1 M:\Documents
Account CCR10838
Submitted_by A. V. Stachulski
Sample_name AVS04-22F
Lab_number 4.03
Staff/Student ID 361286
DEPT2 PMON
22
[ppm]
[rel]

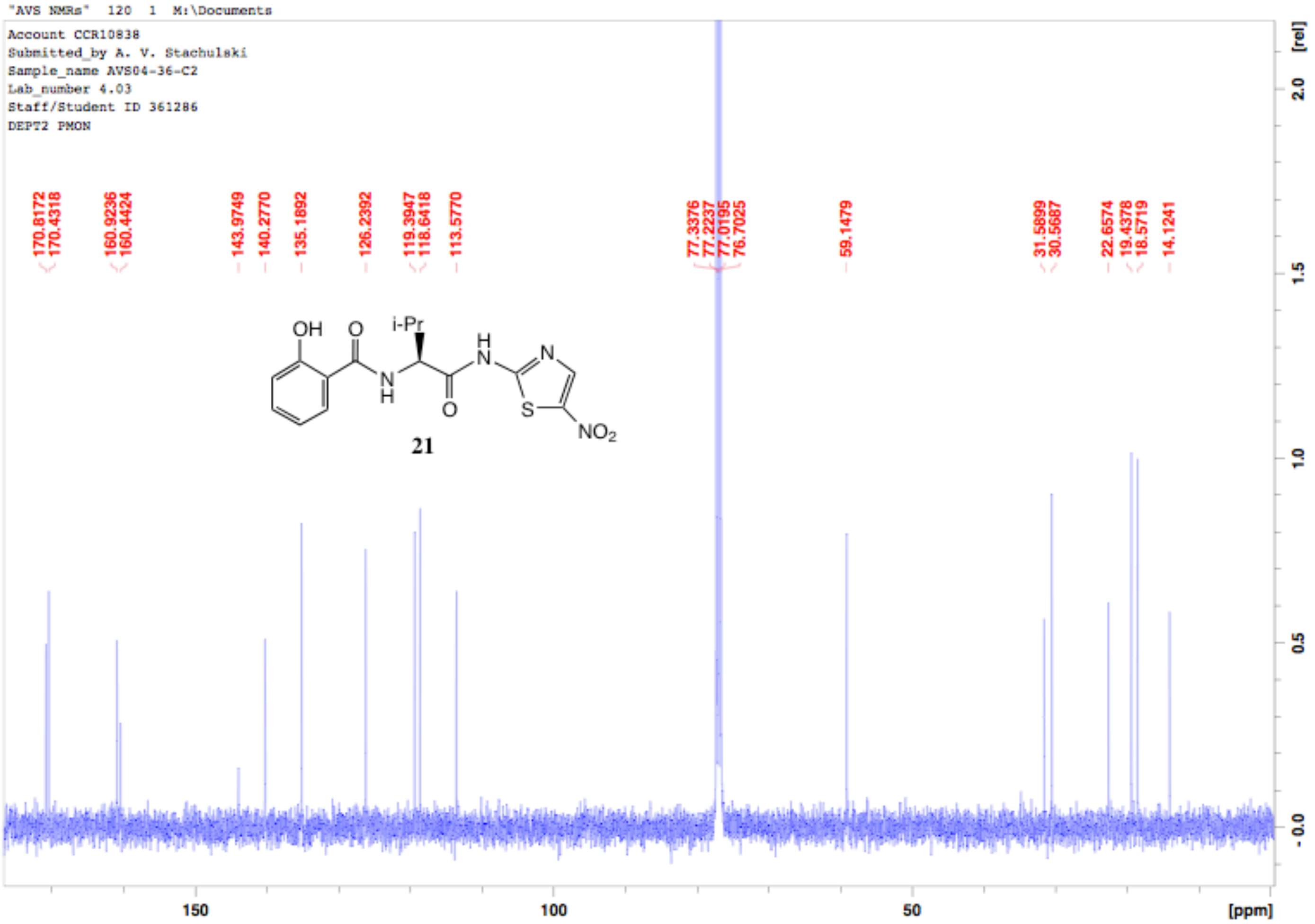

Contains some n-hexane (δ_C 14.1, 22.7, 31.6)

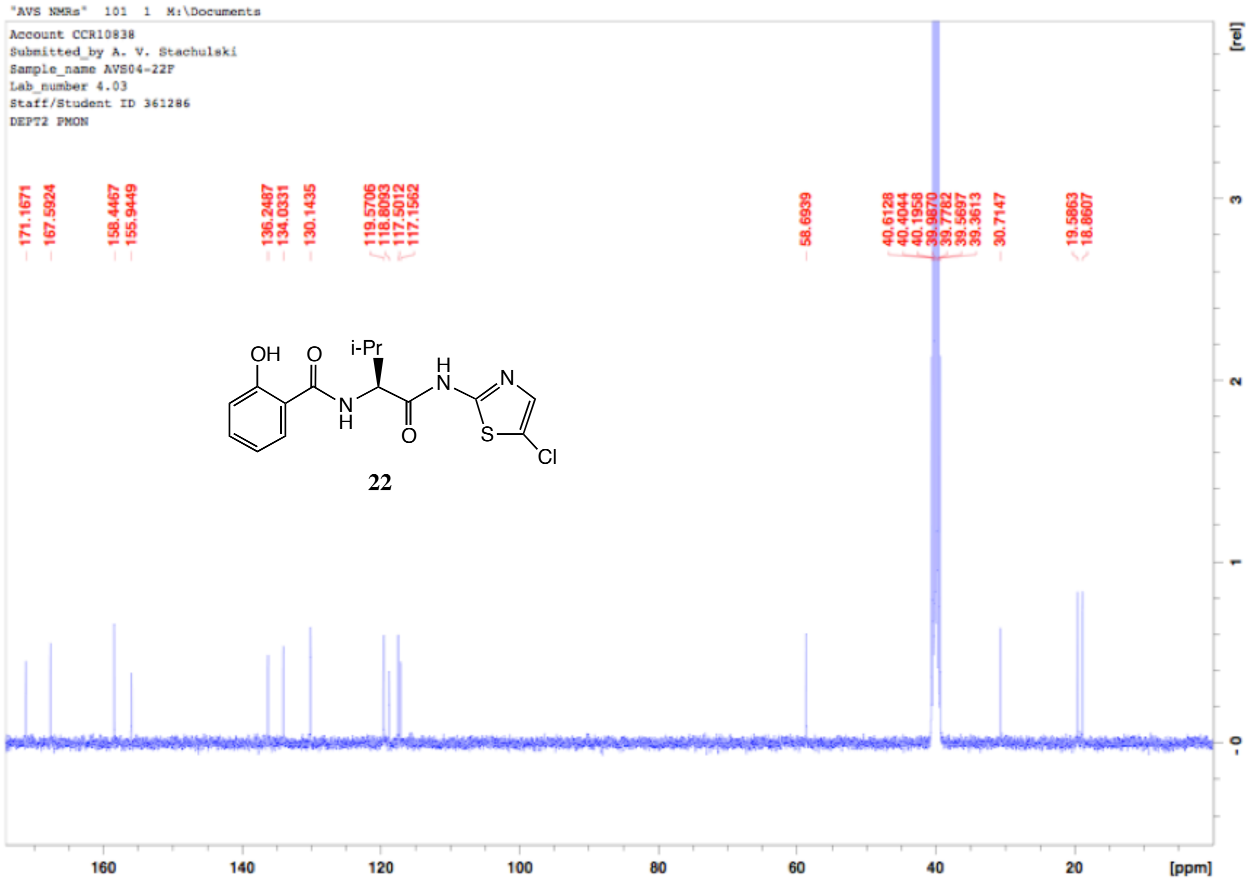
"AVS NMRs" 101 1 M:\Documents
Account CCR10838
Submitted_by A. V. Stachulski
Sample_name AVS04-22F
Lab_number 4.03
Staff/Student ID 361286
DEPT2 PMON
171.1671
167.5924
158.4467
155.9449
136.2487
134.0331
130.1435
119.5706
118.8093
117.5012
117.1562
58.6939
40.6128
40.4044
40.1958
39.9870
39.7782
39.5697
39.3613
30.7147
19.5863
18.8607
OH
O
i-Pr
H
N
N
H
O
N
S
Cl
22
[rel]
[ppm]

2) Full time course NMR spectra for the degradation of **5a**

(a) Stacked spectra over an 18-day period.
Time course ^1H NMR spectra of the aromatic region of **5a** in d_6-DMSO + 10%D_2O**.** Evolution with time over an 18-day period at 298° – 313° K. Sample held in isothermal bath at specified temperature between measurements. Spectra were recorded at the temperature stated in each case. (a) t = zero, 298° K; (b) t= 1 week, 308° K; (c) t = 8 days, 308° K; (d) t = 9 days, 308° K; (e) t = 11 days, 313° K; (f) t = 14 days, 313° K; (g) t = 18 days, 313° K.

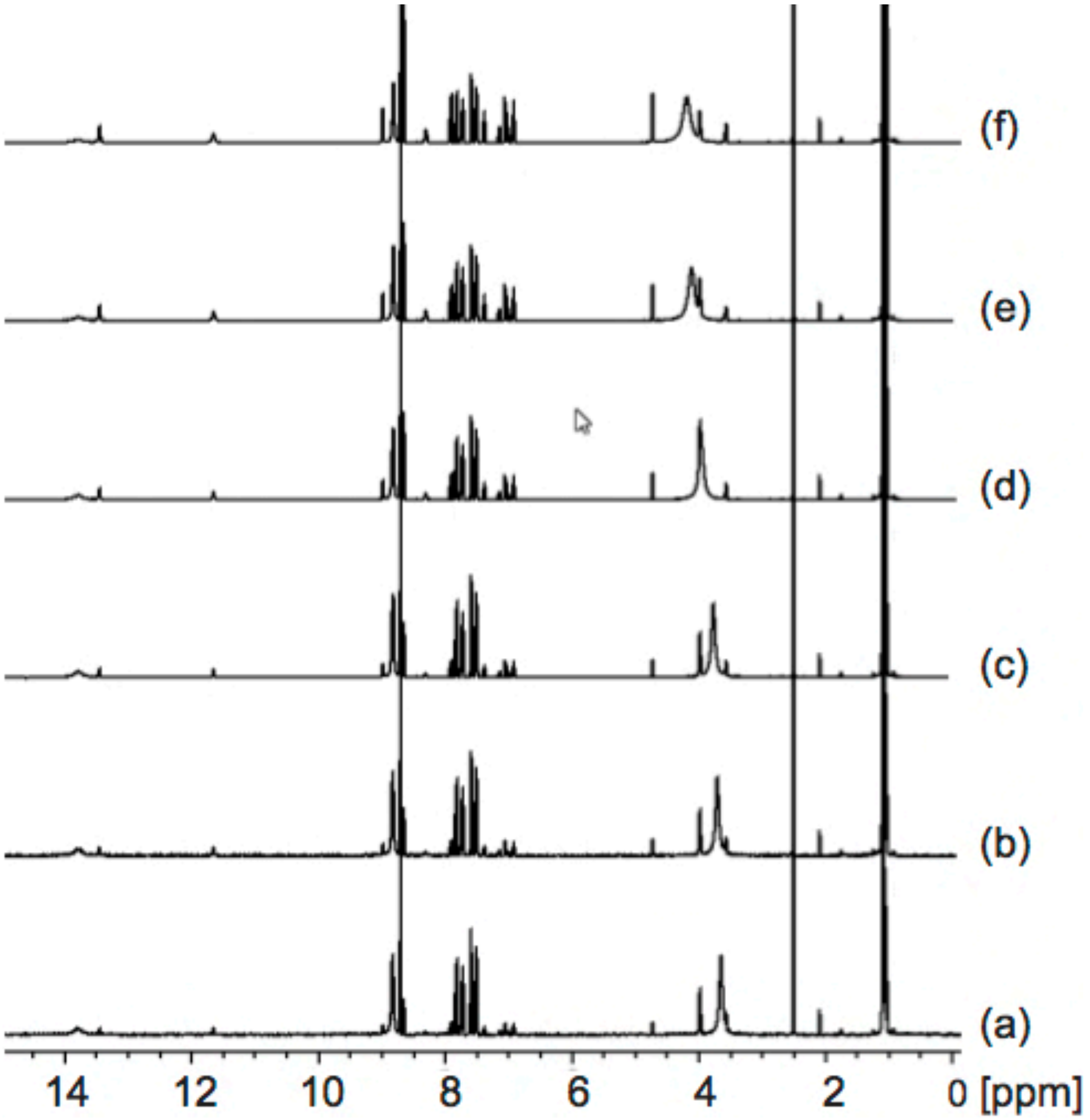

(b) Showing: (a) tizoxanide **3**, (b) prodrug **5a**, (c) rearranged product **8** and (d) the final time point of the degradation sequence (18 h).

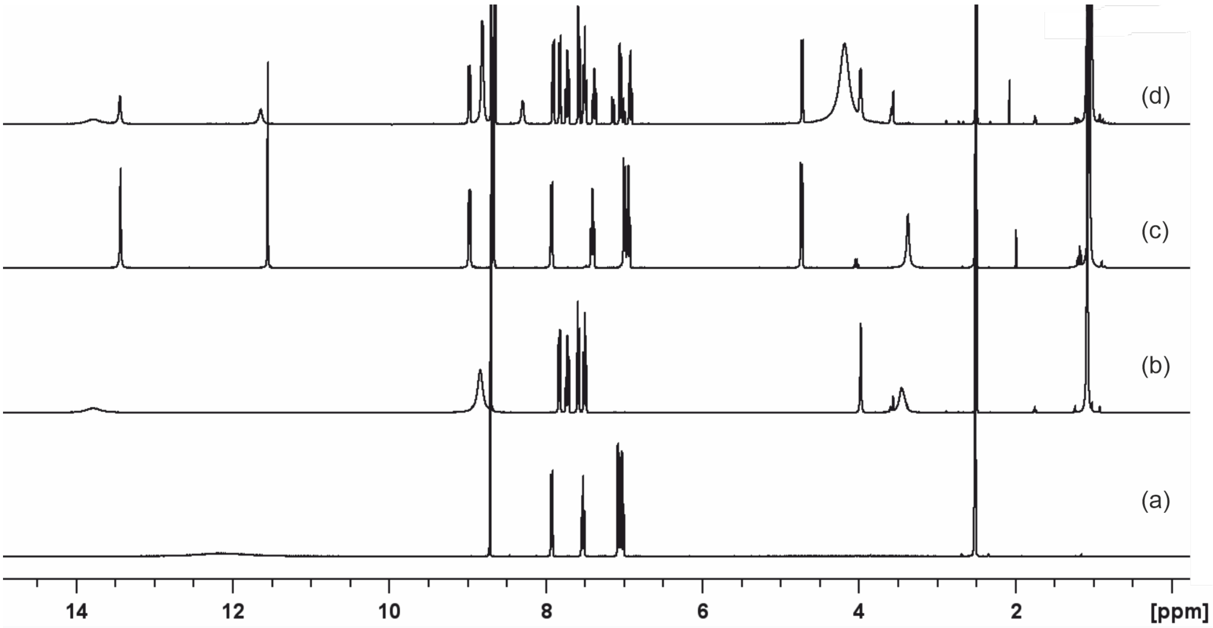

(c) The t-butyl region of the final time point spectrum, showing three signals.

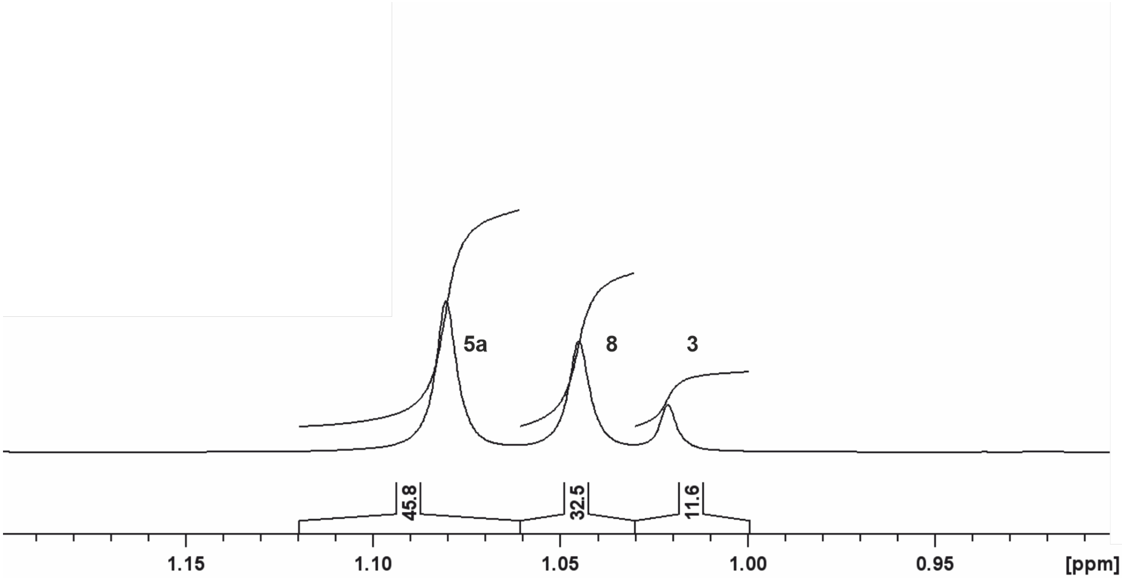

3) Proposed mechanism for the rearrangement of **5a** to **8**, see ref. 19, main text.

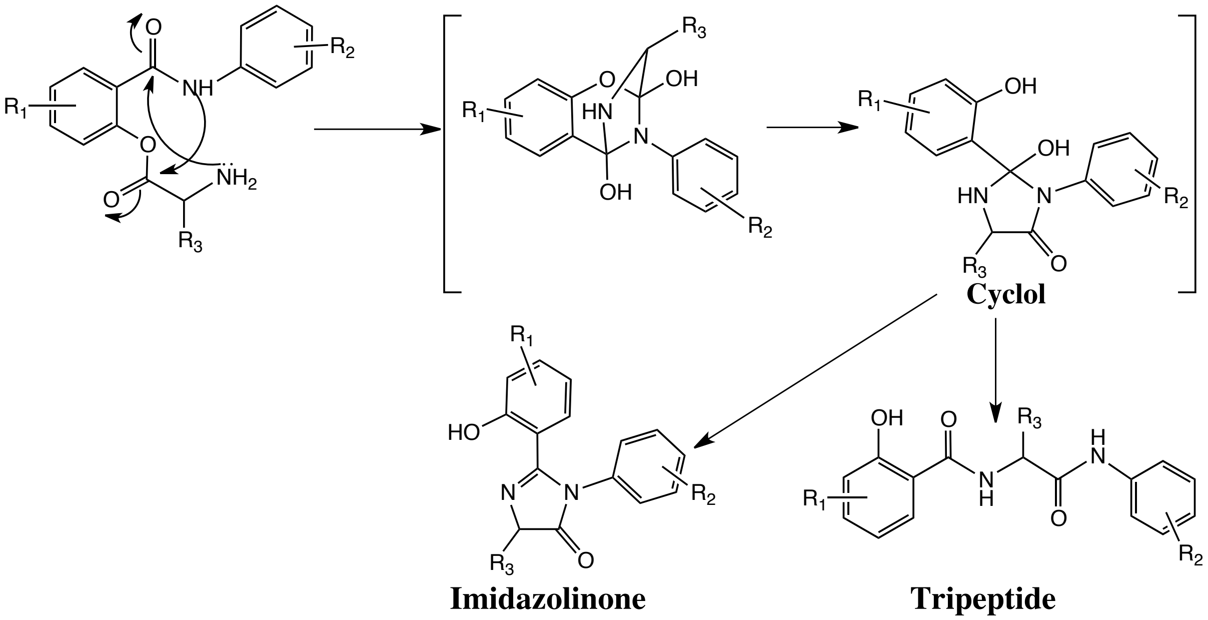

In Vinsova's mechanism, the highlighted **cyclol**, by protonation and dehydration, may lead to the **imidazolinone** shown, cf. **11** in the MS. Alternatively, ring opening leads to the observed **tripeptide**, cf. **8**.

4) Analytical HPLC traces for compounds **8**, **20**, **21 and 22**.

Compound **8**

```
Data File C:\Chem32\1\Data\Shirley\2023-02-21_AVS04-18.D
Sample Name: AVS04-18
```

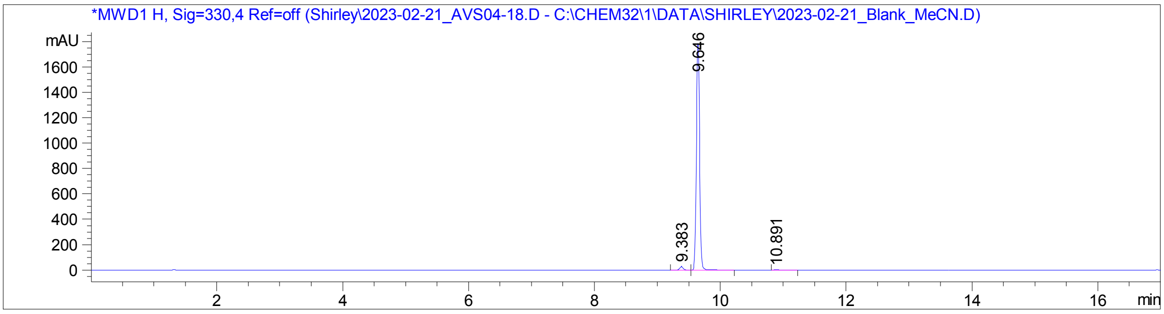

Area purity 98.3%

Compound **20**

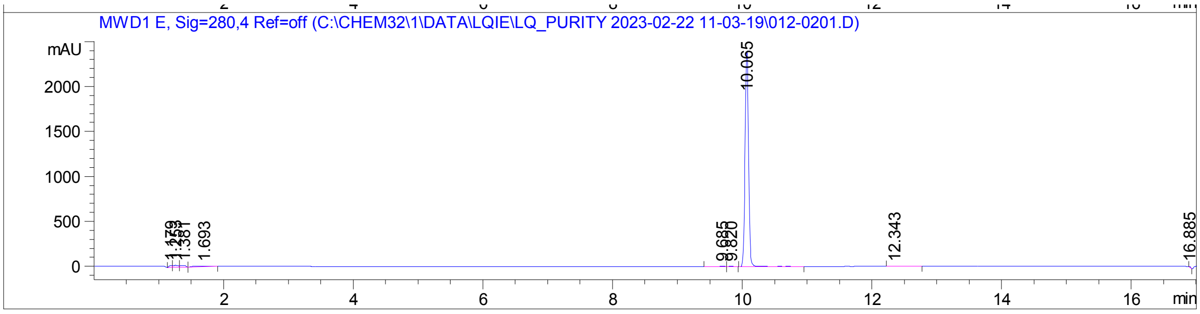

Area purity 99.5%

Compound **21**

```
Data File C:\CHEM32\1\DATA\LQIE\LQ_PURITY 2023-02-22 11-03-19\013-0301.D
Sample Name: AVS-4-33
```

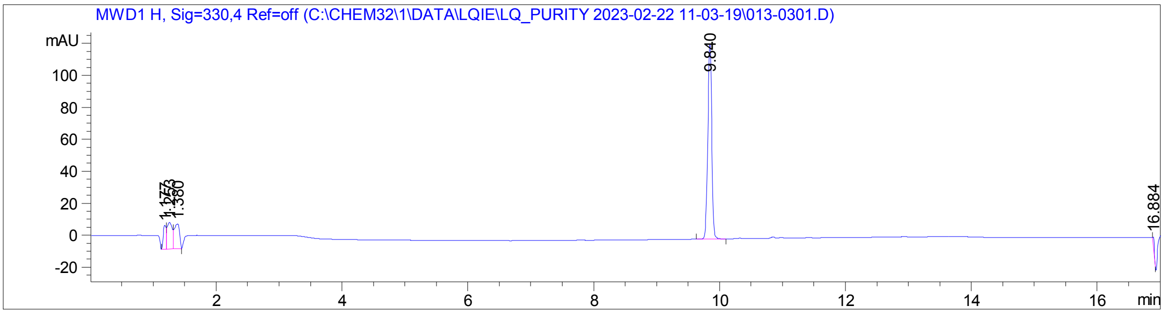

Area purity 97.5%

Compound **22**

```
Data File C:\CHEM32\1\DATA\LQIE\LQ_PURITY 2023-02-22 11-03-19\014-0401.D
Sample Name: AVS-4-36
```

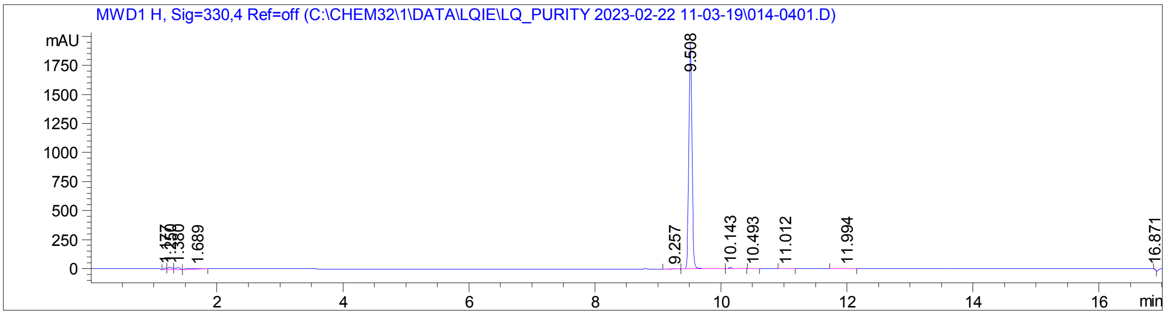

Area purity 98.1%
